# EEG-Based Measures in At-Risk Mental State and Early Stages of Schizophrenia: A Systematic Review

**DOI:** 10.3389/fpsyt.2021.653642

**Published:** 2021-05-04

**Authors:** Andrea Perrottelli, Giulia Maria Giordano, Francesco Brando, Luigi Giuliani, Armida Mucci

**Affiliations:** Department of Psychiatry, University of Campania “Luigi Vanvitelli”, Naples, Italy

**Keywords:** electroencephalogram, first-episode psychosis, first-episode schizophrenia, clinical high-risk, ultra high-risk, frequency bands, microstates, ERPs

## Abstract

**Introduction:** Electrophysiological (EEG) abnormalities in subjects with schizophrenia have been largely reported. In the last decades, research has shifted to the identification of electrophysiological alterations in the prodromal and early phases of the disorder, focusing on the prediction of clinical and functional outcome. The identification of neuronal aberrations in subjects with a first episode of psychosis (FEP) and in those at ultra high-risk (UHR) or clinical high-risk (CHR) to develop a psychosis is crucial to implement adequate interventions, reduce the rate of transition to psychosis, as well as the risk of irreversible functioning impairment. The aim of the review is to provide an up-to-date synthesis of the electrophysiological findings in the at-risk mental state and early stages of schizophrenia.

**Methods:** A systematic review of English articles using Pubmed, Scopus, and PsychINFO was undertaken in July 2020. Additional studies were identified by hand-search. Electrophysiological studies that included at least one group of FEP or subjects at risk to develop psychosis, compared to healthy controls (HCs), were considered. The heterogeneity of the studies prevented a quantitative synthesis.

**Results:** Out of 319 records screened, 133 studies were included in a final qualitative synthesis. Included studies were mainly carried out using frequency analysis, microstates and event-related potentials. The most common findings included an increase in delta and gamma power, an impairment in sensory gating assessed through P50 and N100 and a reduction of Mismatch Negativity and P300 amplitude in at-risk mental state and early stages of schizophrenia. Progressive changes in some of these electrophysiological measures were associated with transition to psychosis and disease course. Heterogeneous data have been reported for indices evaluating synchrony, connectivity, and evoked-responses in different frequency bands.

**Conclusions:** Multiple EEG-indices were altered during at-risk mental state and early stages of schizophrenia, supporting the hypothesis that cerebral network dysfunctions appear already before the onset of the disorder. Some of these alterations demonstrated association with transition to psychosis or poor functional outcome. However, heterogeneity in subjects' inclusion criteria, clinical measures and electrophysiological methods prevents drawing solid conclusions. Large prospective studies are needed to consolidate findings concerning electrophysiological markers of clinical and functional outcome.

## Introduction

Schizophrenia (SCZ) is a severe and complex mental disorder, demonstrating heterogeneity in terms of risk factors, comorbidities, clinical presentations, course, response to treatment, and functional outcome. It approximately affects 26 million people and is currently regarded as one of the leading causes of disability worldwide (1–3). Despite significant advances in the available pharmacological and psychosocial interventions, the impairment in real-life functioning, represents, to date, an unmet need in the care of people suffering from this disorder, with a huge burden on patients, their families, and health-care systems (4–10). Different factors, some related to the illness, others to personal resources, and others to the context, have been demonstrated to contribute to the impairment in functioning (6–8, 10, 11). Among these factors, prolonged periods of untreated psychosis have a role in determining a chronic course of symptoms and a poor functional outcome (12, 13). Therefore, in the last decades much more effort has been invested in the early detection and intervention in schizophrenia, aiming to decrease the risk of deterioration associated to a chronic and relapsing course of the illness.

The first episode of psychosis (FEP) is a crucial stage in the course of schizophrenia, representing the transition from a premorbid to a morbid state. This stage is usually preceded by a “prodromal” period, during which subjects might present gradual and subtle changes in thoughts, perceptions, behaviors, cognition, and functioning (14–16). This period is associated with affective symptoms, social withdrawal, cognitive deficits, attenuated positive psychotic symptoms, and impairment in functioning, which is strongly related to cognitive deficits (17–19). This clinical syndrome has been termed as “at risk mental state” (ARMS) and operationalized criteria were developed to categorize subjects within the clinical high-risk (CHR) or ultra high-risk (UHR) status (14, 20, 21).

It has been demonstrated that within 3 years following the onset of prodromal symptoms, about 18–36% of ARMS subjects make a transition from a premorbid to a morbid state (17). Those ARMS subjects that do not develop a psychosis will present another psychiatric disorder or persistent attenuated symptoms, while only about 14% will have symptomatic remission (22–24).

It seems, therefore, crucial to promptly detect early stages of psychosis, including the state of vulnerability and the onset of psychosis, in order to implement adequate interventions, reduce the rate of transition to psychosis, as well as the risk of further progression to deteriorating stages and impairment in real-life functioning.

In order to characterize subjects that are at risk to develop a psychosis and those with a FEP, research efforts have been directed toward the establishment of the neurobiological underpinnings of these early illness stages, excluding the bias of chronicity, medications, and institutionalization, present in subjects with chronic schizophrenia (18, 25–28). These neurobiological correlates can be investigated effectively with electroencephalography (EEG). Indeed, EEG represents a good and appropriate technique to analyze the neurophysiology of both normal and psychotic experience and behavior, based on an integrative, complex and *in-vivo* model of the brain (29–33). In addition, this technique is non-invasive, and, in comparison to other imaging techniques, such as functional magnetic resonance imaging (fMRI), EEG has the advantages that it is more flexible in study design, has lower costs and it exhibits a superior temporal resolution. Furthermore, through source analysis methodology, such as Low Resolution Electromagnetic Tomography (LORETA), it is possible to obtain information about brain areas from which the neuronal activity is generated (34, 35).

EEG-based measures can be categorized schematically into three categories. The first one considers the oscillatory nature of neuronal activity. This usually involves dividing the continuous recorded EEG signal into its different frequency bands (delta, theta, alpha, beta, and gamma) (36). These oscillatory rhythms can be recorded while subjects are in state of relaxation and without any external stimulation or, alternatively, during sensory stimulation or while performing a task.

The second category is represented by microstates (MS), defined as brief periods during which global electrical brain activity remains semi-stable. These transient periods of stability last between 80 and 120 ms (37, 38). Each microstate is classified on the basis of its corresponding EEG scalp potential map (39, 40). Microstates are hypothesized to be the most basic instantiations of human neuronal functions and are thus nicknamed as “the atoms of thought.”

Finally, the third category is constituted by event-related potentials (ERPs), which reflect the neuronal response following a specific sensory, cognitive, or motor event (32, 41). These EEG indices can manifest as positive and negative voltage deflections, waves, or components with a precise temporal correlation to the onset of a specific event (42).

A vast EEG literature has documented different abnormalities of neuronal activity in subjects with chronic SCZ, as compared to healthy controls (HCs) (29, 33, 43–49). In particular, several studies have consistently reported alterations in the activity of the whole spectrum of frequency bands (49–53), changes in MSs topography and/or other parameters (46, 47, 54) and a reduction of amplitude in ERPs, such as N100 (55–58), mismatch negativity (MMN) (59, 60), and P300 (33, 57, 61). Furthermore, these alterations have been related to the severity of symptoms, as well as cognitive and functional impairment in schizophrenia. For instance, aberrant frequency bands activity has been associated to cognitive deficits and positive and negative symptoms (50–53); alterations in MS parameters to negative symptoms, hallucinations, and duration of illness (46, 47, 54); reductions in N100 amplitude to auditory hallucinations and attention deficits (55, 57, 58); deficits in MMN to positive symptoms and functioning impairment (57, 59, 60); P300 to neurocognitive impairments and negative symptoms (57, 61).

In the last decades, a large number of studies has highlighted how some of the abnormalities of EEG indices, reported in subjects with chronic SCZ, can also be observed in the ARMS and prodromal or early phases of schizophrenia. Previous reviews on this topic (25, 44, 62–68) have considered either at-risk/prodromal states or early stages of schizophrenia or only one specific electrophysiological index.

In the light of these observations, the aim of the present study is to review the current evidence concerning abnormalities of electrophysiological indices, including all three categories of indices mentioned above, in both CHR/UHR and FEP subjects.

## Methods

### Study Design

The Preferred Reporting Items for Systematic Reviews (PRISMA) statement has been followed to design and conduct the systematic review (69).

In brief, we performed a comprehensive literature search on abnormalities of EEG indices in the early stages of schizophrenia (FEP and CHR/UHR subjects).

In order to facilitate the comprehension of the reader, we clarify the terms that we used in the manuscript. The term FEP encompasses both affective-spectrum disorders (bipolar disorder and major depressive disorder with psychotic features) and schizophrenia spectrum disorders (schizophrenia, schizoaffective disorder, and schizophreniform disorder). The term first episode of schizophrenia (FES) is used to indicate exclusively those subjects who, although falling also within the FEP categorization, are specifically at the onset of a schizophrenia spectrum disorder. Therefore, in the present manuscript, whenever studies did not specify the characteristics of the FEP sample or if the sample was heterogeneous and included subjects with either a first-episode of a schizophrenia-spectrum disorder or of an affective disorder (i.e., bipolar disorder or major depressive disorder with psychotic features), we indicated the sample as “FEP.” Conversely, we indicated as “FES” the samples for which the authors clearly specified a diagnosis of a schizophrenia-spectrum disorder. Through the whole manuscript with the term “high risk” (HR) we will refer to subjects in the ARMS or prodromal stages of the illness. The original nomenclature of the study samples will be kept within tables reporting the description of the studies included in the present review.

### Articles Research Strategy

A systematic literature search was conducted in three electronic databases: PubMed, Scopus, and PsychINFO on 13th July 2020 with no time limit and with English language as the only selected filter, in order to ensure that it was as comprehensive as possible (Table 1).

**Table 1:** Systematic search strategies.

**Database**	**Search syntax**	**Number of retrieved documents**	**Date of search**
PubMed	(EEG OR electroencephalography OR “EEG microstate” OR “dipole source localization” OR sLORETA OR LORETA OR eLORETA OR ERP OR “event-related potential” OR “spectral analysis” OR “frequency domain analysis” OR “spectral band” OR “neural oscillations” OR “spectral power” OR N100 OR N1 OR MMN OR “mismatch negativity” OR P300 OR P3a OR P3b OR “event-related” OR “evoked potential” OR “evoked-response”) AND (“ultra-high risk psychosis” OR “clinical high risk psychosis” OR “prodromal psychosis” OR “first episode schizophrenia” OR “first episode psychosis” OR “early onset schizophrenia”) Filters: Languages, English Search in [All fields] No time limit	220	13.07.2020
Scopus	(EEG OR electroencephalography OR “EEG microstate” OR “dipole source localization” OR sLORETA OR LORETA OR eLORETA OR ERP OR “event-related potential” OR “spectral analysis” OR “frequency domain analysis” OR “spectral band” OR “neural oscillations” OR “spectral power” OR N100 OR N1 OR MMN OR “mismatch negativity” OR P300 OR P3a OR P3b OR “event-related” OR “evoked potential” OR “evoked-response”) AND (“ultra-high risk psychosis” OR “clinical high risk psychosis” OR “prodromal psychosis” OR “first episode schizophrenia” OR “first episode psychosis” OR “early onset schizophrenia”) Filters: Languages, English Search in [Title/Abstract/Keywords] No time limit	221	13.07.2020
PsychINFO	(EEG OR electroencephalography OR “EEG microstate” OR “dipole source localization” OR sLORETA OR LORETA OR eLORETA OR ERP OR “event-related potential” OR “spectral analysis” OR “frequency domain analysis” OR “spectral band” OR “neural oscillations” OR “spectral power” OR N100 OR N1 OR MMN OR “mismatch negativity” OR P300 OR P3a OR P3b OR “event-related” OR “evoked potential” OR “evoked-response”) AND (“ultra-high risk psychosis” OR “clinical high risk psychosis” OR “prodromal psychosis” OR “first episode schizophrenia” OR “first episode psychosis” OR “early onset schizophrenia”) Filters: Languages, English; Species, Human Search in [All Fields] No time limit	173	13.07.2020

The following combination of search terms was used:

(EEG OR electroencephalography OR “EEG microstate” OR “dipole source localization” OR sLORETA OR LORETA OR eLORETA OR ERP OR “event-related potential” OR “spectral analysis” OR “frequency domain analysis” OR “spectral band” OR “neural oscillations” OR “spectral power” OR N100 OR N1 OR MMN OR “mismatch negativity” OR P300 OR P3a OR P3b OR “event-related” OR “evoked potential” OR “evoked-response”) AND (“ultra-high risk psychosis” OR “clinical high risk psychosis” OR “prodromal psychosis” OR “first episode schizophrenia” OR “first episode psychosis” OR “early onset schizophrenia”).

The search terms were selected to include both general terms related to EEG research and more specific indices (such as specific ERPs) that have been consistently investigated in research papers attaining to schizophrenia. In addition, reference lists were hand-searched to identify additional publications missed by the search strategy.

### Selection Process and Criteria

Firstly, any duplicate from the combination of the three databases was excluded. The remaining articles were included in the systematic review only if they met the following criteria:

*Inclusion criteria*

1. meta-analysis, reviews, case-control studies concerning the abnormalities of EEG indices in the at-risk, prodromal and early stages of schizophrenia;
2. studies carried out in humans;
3. studies published in English;
4. studies that included at least one group of subjects during their at-risk, prodromal or early stages of illness, compared with a healthy control group;

*Exclusion criteria*

1. books chapters, comments, editorials, case reports/case series, theses, proceedings, letters, short surveys, notes;
2. studies irrelevant to the topic;
3. unavailable full-text.

If the studies included in the present review reported data concerning differences between HR subjects who made the transition to psychosis (HR-T) from those who did not (HR-NT), as well as data concerning differences between subjects at at-risk/prodromal/early stages of psychosis and subjects with chronic schizophrenia, these data have been also incorporated in the present paper.

Two researchers (AP, FB) independently screened for eligibility all the articles by titles and abstracts and then proceeded to read the full text. Discrepancies in the selection of the eligible articles have been discussed in advance with the whole group and were resolved by discussion and consensus.

### Data Extraction

We recorded the following variables from each included article: author/s, year of publication, EEG index evaluated, study population, assessment instruments for diagnosis and EEG data results (Supplementary Tables 1–3). Given the heterogeneity of experimental paradigms and considered variables in the eligible studies, we did not plan to carry out a meta-analysis.

## Results

### Characteristics of the Included Studies

**Figure 1:**
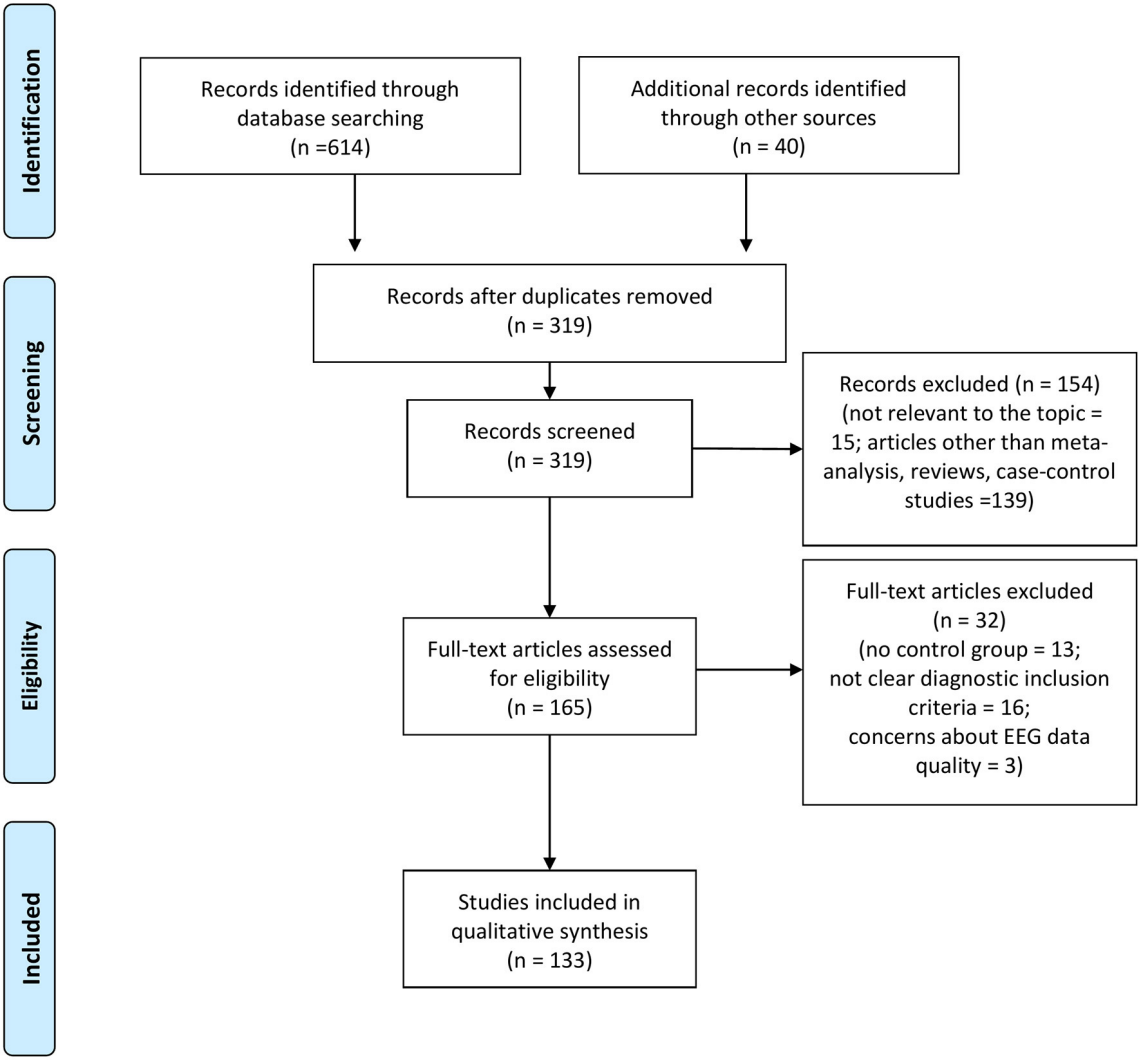
PRISMA flow chart of included studies.

The combined outcome of the three databases results yielded a total of 614 records (Figure 1). In addition, 40 studies were included by hand search. Of the total studies, 335 were duplicates, leaving 319 articles. After reading the titles and abstracts, 154 of these were excluded because they were not relevant to the topic of the review or because they were articles other than meta-analysis, reviews and case-control studies. The full text of the remaining 165 studies was examined in more detail. It appeared that 32 studies did not meet the inclusion criteria due to methodological discrepancies (i.e., no control group included, no clear EEG data reported, no clear explanation of diagnostic criteria of sample). Therefore, a total number of 133 studies were finally identified as eligible for inclusion in the current review (Figure 1).

### EEG Frequency Bands

The EEG raw signal can be decomposed into five main oscillatory rhythms or broad frequency bands, namely delta (0.5–4.0 Hz), theta (4–8 Hz), alpha (8–13 Hz), beta (13–30 Hz), and gamma (30–100 Hz) bands (43, 62, 70). These bands can be investigated during both resting-state condition and during sensory stimulation or task performance. In the context of frequency bands analysis, different measures have been considered, such as the power and the source localization of each frequency band activity and indices of coherence, synchronization, and neuronal connectivity. The studies included for this section are reported in Supplementary Table 1.

#### Delta Band

Delta activity, characterized by a spectral bandwidth 0.5–4.0 Hz, is the predominant slow wave in states of unconsciousness, such as sleep and anesthesia. Furthermore, following external stimulation, synchronization of evoked responses in delta band across neuronal regions, plays also a role in motivational, emotional, and cognitive functions (71, 72).

Resting-state EEG data showed consistent increases in delta band power in subjects with chronic SCZ, as compared to healthy controls (33, 43, 72). Therefore, different studies have been conducted aiming to verify whether this abnormality is present since the early phases of the illness.

Findings concerning delta band during resting-state in the early phases of schizophrenia are controversial, with some studies reporting no abnormality in delta power (73) or synchrony (74), while others finding differences between these subjects and HCs in power (75–79) or functional connectivity (80) within this band activity.

Specifically, studies during a resting-state condition that found abnormalities in delta band in FES compared to HCs, have reported a higher power (75–77). Furthermore, additional studies and analyses also showed a lower delta amplitude peak (77), an excess of delta activity in prefrontal areas (78), and a distributed hypo-synchronization of delta activity between cerebral regions, mostly observed in parietal areas as suggested through LORETA analysis (78, 80). One longitudinal study in FEP subjects, with a 1-year follow-up, which also considered electrode location for its analysis, found that higher baseline delta activity at posterior regions predicted improvement in positive symptoms after 1 year, while lower values in the same band at frontal regions were associated with the amelioration of negative symptoms (76).

An increase in delta power in frontal areas, considering scalp electrode location, has been observed also in HR-T compared to HR-NT and to HCs, while no difference between HR-NT and HCs was recorded (79).

Three studies investigated delta activity while subjects performed a task (81–83). In one study, FES subjects, as compared to HCs, had a decrease in the occurrence of anterior–to-posterior propagation of delta waves during auditory and visual tasks (82). A second study, carried out in FEP subjects, demonstrated a workload-dependent increase of the duration of delta oscillations during a working memory task (81) not observed in HCs. This was revealed by longer delta oscillation cycles in FEP during the performance of tasks of increasing difficulty. Another study reported no difference between FES and HCs in terms of delta functional connectivity and its topological properties assessed while subjects performed an executive functioning task (83).

#### Theta Band

Theta oscillatory activity (4–8 Hz) seems to be involved in the orchestration of several cognitive processes, such as working memory, detection of new sensory stimuli and attentional control (84). It has been reported that subjects with chronic SCZ present different abnormalities in this frequency range, often characterized by excessive frontal theta activity (33, 43, 85, 86).

Inconsistent findings have been reported in studies investigating abnormalities of the theta band during a resting state condition in FES, FEP, and HR subjects, as compared to HCs. In particular, some studies reported no significant difference in the theta band activity between HCs and FEP (87), FES (73, 76, 77), or HR (87) subjects. Other studies reported an increase in theta band activity in FES subjects compared to HCs (75, 88) during resting conditions. The increase in theta activity in FES was associated with the severity of negative symptoms (88) and this alteration was observed also in HR-T if compared to HR-NT and HCs (79). Abnormalities in theta oscillations were also found in studies that performed a source localization analysis through LORETA (78, 89). These studies found a decrease of theta activity specifically in the anterior regions in FEP (89) and FES subjects (78) as compared to HCs. Furthermore, a complex LORETA study investigating theta-gamma amplitude phase coupling (regulation of the gamma band activity depending on the phase of theta band activity) showed an alteration in this index in a FES sample in the posterior cingulate cortex (90). Some studies investigated the neural connectivity during resting-state and found a decrease in the global field synchronization (74), or an increase in the synchronization between posterior cingulate cortex, cuneus, and precuneus (80) or an increased connectivity between brain networks (91–93) in the theta band in FES subjects, as compared to HCs. Connectivity values were also related to clinical symptoms (91), worse verbal memory (92), and processing speed (93). On the other side, a study that included also HR subjects, did not detect any significant difference between these subjects and HCs in theta functional connectivity (92).

Abnormalities in theta activity were reported also during task performance (83, 88, 94–96). In particular, task-related abnormal theta activity was detected during a processing speed (94), an arithmetic (88) or an auditory task (95) in FES (88, 95) and FEP subjects (94). In addition, two studies highlighted brain network dysfunctions in theta band in FES as compared to HCs, as suggested by abnormalities in neuronal information transmission during an executive functioning task (83) and a reduction of the “small-world network” index (96), a parameter assessing cerebral networks topology and the efficiency of neuronal signaling processing. Conversely, the latter index has not been found to be different between HR subjects and HCs in another study (96).

#### Alpha Band

Alpha oscillation is one of the most prominent neuronal rhythm in the adult human brain, both during resting-state condition and task performance, and it is characterized by a frequency spectrum ranging from 8 to 12 Hz (97). Neuronal oscillations within this frequency band play a pivotal role in cognition, consciousness, sensorimotor and emotional processes (98). In subjects with SCZ, a decrease in absolute power during resting-state conditions (43) and disruptions in temporal coherence in evoked oscillations during sensory stimulation and cognitive tasks have been reported (99).

During at-risk and early phases of the disorder, alpha activity shows already alterations in its features. Several studies revealed a reduction in alpha frequency activity (75, 88) in diverse and widespread cerebral areas including frontal (73, 89, 100), parietal (73, 78, 100), temporal (78, 100), and occipital (100) regions in FES and FEP subjects compared to HCs, as assessed through LORETA. Conversely, other studies found no significant difference in the alpha power between HCs and FEP (87, 100), FES (73, 76, 77), and HR (87) subjects. Furthermore, no robust differences were detected in alpha power when HR-T, HR-NT, and HCs were compared (79).

Inconsistent results have been reported also for EEG connectivity indices recorded during rest. One study (74) did not find any difference between FES subjects and HCs in terms of global field synchronization. Conversely, other studies reported abnormalities in connectivity-related indices in alpha band, such as a lower coherence (101), a generalized hypo-synchronization across cerebral regions (80) and lower phase-lag index (PLI) (93) in FES subjects, compared to HCs. Yet, another study reported significantly higher PLI values of alpha in FES and HR subjects compared to HCs (102), contradicting previous results (93).

In studies focusing on stimuli or task-related activity, it was shown that alpha activity, evoked by error commitment, was significantly increased in FES subjects (94) compared to HCs. Task-related connectivity studies focusing on alpha band found either no difference in signal complexity and brain network communication (83), or a decrease in coherence (101) in FES compared to HCs. Finally, a reduction in alpha desynchronization (103) and a higher clustering coefficient of the alpha band (96) were detected in HR as compared to HCs.

#### Beta Band

Beta oscillation (12–30 Hz) has been studied mostly in relation to sensorimotor behavior and cognitive processes, such as working memory and top-down regulation of attention (104, 105). In schizophrenia, beta-band abnormalities manifest as increased activity in resting-state (106, 107) and in relationship to perceptual integration (108).

Five studies found no differences between FEP or FES subjects, as compared to HCs in the power of the beta frequency band during a resting-state condition (73, 75–77, 109). Conversely, three studies found differences in the beta power between FEP (89) or FES (78, 88) subjects and HCs. In particular, these studies found an increase in beta power in FEP and FES, which was associated with the severity of negative symptoms (88) and localized mainly in the right parietal area (78) and frontal gyrus (89), as revealed by LORETA analysis (78, 89). Focusing on connectivity measures during rest, FES subjects, compared to HCs, showed a lower EEG coherence (109), as well as a generalized hypo-synchronization in the beta band (80). However, a study reported no significant difference between FES subjects and HCs in the global field synchronization of the beta band (74).

In task-related recordings, an increase in the power (88) and a reduction in coherence (101) were reported for the beta band in FES subjects, compared to HCs. The decrease in coherence was also associated to the severity of positive symptoms in one of the studies (101). Finally, no abnormal mean value was recorded through the evaluation of path length of networks activity within the beta band, while subjects performed an executive functioning task (83).

#### Gamma Band

Gamma activity represents the fastest oscillations (30–100 Hz) of the spectrum, and these fast waves are generated through the synchronized activation of pyramidal neurons located in the cerebral cortex (62, 110). Gamma band oscillations have been linked to a vast variety of cognitive and perceptual integration processes (110) and showed several abnormalities in subjects with SCZ, often associated to impairments in neurocognitive functions (111).

A systematic review has already summarized how abnormalities in gamma band have been vastly reported in FEP and HR subjects (62).

During resting state conditions, an increase in gamma power was reported in FES (112), FEP (89), and HR-T subjects (113) vs. HCs, mainly located in the frontal regions (89, 113) when LORETA analysis was implemented. However, no difference in resting-state gamma activity was observed in three other studies (88, 91, 109), between FES subjects and HCs. As regard to connectivity indices during rest, a generalized hypo-synchronization (80), an increase in connectivity (91), a decrease in coherence (101, 109) and a reduced phase lag index (PLI) (93) in the gamma band were found in FES subjects, as compared to HCs.

During sensory stimulation or the performance of a task, different results have been reported. In particular, several studies reported abnormalities in the evoked responses (mainly decrease in evoked gamma), synchronization (reduction or a delay in the synchronization) and connectivity (generally a reduction in functional connectivity) of gamma activity during the performance of memory (81, 114), cognitive control (115), emotion processing (116, 117), or auditory tasks (118–122) both in FES (114, 115, 117–122) and FEP (81, 116) subjects, as compared to HCs. Furthermore, alterations in gamma synchrony were also related to social cognition in one study (117), while another one, employing a longitudinal design, highlighted that the improvement in positive symptoms in FES subjects was related to the increase in gamma synchrony (121). Findings concerning the impairment in synchronization of gamma during the performance of a task in HR subjects are discrepant. Perez et al. (123) reported a decrease in gamma evoked response. A similar result was also reported in a study using an auditory task and multimodal recordings (simultaneous fMRI and EEG), which showed that HR subjects presented a reduction in gamma evoked response, characterized mainly by deficits in activity of the auditory, thalamus, and frontal brain areas (124). Furthermore, although Oribe et al. (118) found a decrease in the coherence of auditory-evoked gamma activity, assessed through phase-locking factor (PLF) in FES, as in the study by Leicht et al. (122), no abnormality was found in HR subjects, as compared to HCs, indicating that this alteration might be linked to more advanced illness phases (118).

Another way to investigate gamma activity is through the analysis of the auditory steady-state response (ASSR), which involves the presentation of auditory stimuli at high frequencies with subsequent entrainment of oscillatory activity at the same frequencies. In a study involving auditory stimulation at 20, 30, and 40 Hz, the FEP group had significantly reduced phase locking and evoked power compared to HCs (125) for gamma ASSR elicited with 30 and 40 Hz stimuli. In this study, higher phase locking was related to more severe positive symptoms (125). Furthermore, studies found a decrease in the evoked gamma power (125), inter-trial phase coherence and spectral perturbation of ASSR (126, 127) in FES subjects compared to HCs. It was also noticed that these alterations in ASSR activity were related to general psychopathology and attentional deficits (126).

In a study with subjects at-risk, no difference was detected in ASSR-evoked gamma power or PLF between the HR subjects and control groups (128), while in another study a decrease of inter-trial phase coherence (ITC) and event-related spectral perturbations in late phases of ASSR was found in HR compared to HCs (126).

### EEG Microstates

The microstates (MS) are EEG-based measures that define the global functional state of the brain by its momentary scalp electric field configuration (39, 129, 130). There is a small set of prototypical MS configurations, which constitutes a basic repertoire of brain functional states: MS-A, MS-B, MS-C, MS-D. In particular, MS-A and MS-B have been found to be associated to BOLD signal within fronto-temporal and occipital regions, areas belonging to the phonological and visual networks, while MS-C and MS-D have been linked to cingulate cortex, right superior and middle frontal gyri, the right superior and inferior parietal lobules, regions involved in the default mode, salience and attention networks (131, 132). Evidence of EEG MS alterations in subjects with SCZ has been widely reported (25, 46, 47, 133). However, few studies investigated these alterations in the early phases of the illness. The studies included in the current review are summarized Supplementary Table 2.

FES subjects, as compared to HCs, showed a reduced duration of MS-B (134) and D (54, 134), an increased occurrence of MS-A (54, 134) and C (134), as well as an increased contribution of MS-A (134) and D (54). The reduced duration of MS-D has been found to correlate with the severity of paranoid symptomatology (54). In addition, the MS syntax A → C → D → A, which predominated in HCs, was reversed in FES (A → D → C → A) (134). Finally, the topography differed between FES and HCs, with FES showing a stronger left and anterior activity of MS-B (134) and central activity of MS-D (54).

Some of the above-mentioned abnormalities have been reported already in HR subjects (135, 136). In particular, HR, as compared to HCs and SCZ subjects, showed an increase in the contribution and occurrence of MS-A; they also showed a reduction in the contribution of MS-B, as compared to SCZ. The aberrant spatial configuration of MS-B, which exhibited a stronger activity in the left posterior cingulate in SCZ subjects, was displayed to a lesser extent also in HR, as compared to HCs (136). In individuals with 22q11.2 deletion syndrome (22q11DS), known to have a 30-fold increased risk to develop schizophrenia, an increased presence of MS-C vs. HCs, was found and was associated with hallucinations (135).

### ERPs Studies

A variety of ERPs, related to sensory-perceptual and cognitive events, have been utilized in schizophrenia research due to their high sensitivity to transient changes in neuronal activity. The following paragraphs report if alterations in P50, N100, mismatch negativity (MMN), P300, and N400 components are detectable already in FEP and HR subjects. These studies are summarized in Supplementary Table 3.

#### P50

P50 is an early event-related positive potential, which is recorded ~50 ms after the presentation of an auditory click stimulus. In a paired-click paradigm, characterized by two subsequent stimuli, a reduction in P50 response after the second stimulus (S2), compared to P50 recorded after the first one (S1), is expected. This is the outcome of a regulatory mechanism known as sensory gating, which is assessed using the P50 ratio (S2-P50 divided by S1-P50). When subjects do not show a diminished response to the second stimulus, a defect in sensory gating is likely to have occurred. Several articles reported an increase in the P50 ratio and difference in SCZ, suggesting a deficit in sensory gating (137–142).

A recent meta-analysis reported a consistent impairment in P50 sensory gating in FEP subjects, as compared to HCs, and highlighted that this deficit had a similar magnitude to the one reported in subjects with chronic SCZ (44). Different studies found a deficit in sensory gating, as measured with the P50 ratio, in FEP (143–146), FES (147, 148), and HR subjects (143, 148, 149), as compared to HCs. The grade of the impairment seemed to be influenced by the clinical presentation of the illness (no sensory gating deficit has been detected in FES subjects during the post-acute phase, after improvement of positive symptoms) (147). Some other studies did not find an impairment in sensory gating in FEP (150–153), FES (154, 155), and HR (150, 151, 154, 156, 157) subjects. Furthermore, a study involving the innovative implementation of machine learning (ML) to distinguish FES from HCs with P50-related measures (amplitude and ratio), in addition to other neuroimaging and clinical evaluations, highlighted that this EEG-index did not contribute significantly to the discrimination performed by the mathematical model (158).

In a study involving FES and HR subjects, which analyzed P50 through LORETA, it was shown that both groups presented differences in the brain functional networks sustaining this ERP, and that these two groups actually showed similarities, suggesting compromised gating already at at-risk stages (154). In particular, FES subjects showed a greater connectivity in the right superior frontal gyrus and right insula, while HR subjects had a greater connectivity in the paracentral lobule and the middle temporal gyrus, as compared to HCs (154).

#### N100

N100 is one of the largest auditory and visually evoked ERP and can be visualized as a negative deflection peaking between 80 and 120 ms after the stimulus onset, with its maximal amplitude recorded over fronto-central leads (159). Subjects with chronic schizophrenia, compared to HCs, show a robust reduction in the amplitude of N100 and in N100-related measures of sensory gating (56, 159, 160). Findings concerning the presence of abnormalities of N100 in the early stages of psychosis are controversial. In particular, some studies demonstrated that FEP subjects, compared to HCs, showed a reduced N100 amplitude both during visual (161) and auditory paradigms (162–164), while other studies did not detect any N100 amplitude impairment in FEP subjects (152, 165–167). In HR subjects one study reported a reduction in N100 amplitude (163), while most of the studies reported similar values of N100 amplitude in HR subjects and HCs (161, 165, 168–171).

When sensory gating was assessed through the presentation of two subsequent stimuli, the N100 amplitude difference (S2-S1) (143, 150) and the N100 gating ratio (S2/S1) (143, 150, 152) showed a significant increase in FEP (143, 150, 152) and HR subjects (143, 150), suggesting an impairment in the processing of redundant stimuli (143, 150). Conversely, some studies did not find any statically significant difference in N100 amplitude (152) or N100 gating ratio (151) in FEP (150–152) and HR subjects (151), as compared to HCs.

N100 latency did not differ in FEP or in HR subjects compared to HCs in any of the studies cited above that analyzed this variable (143, 161–163, 167, 168, 170, 172).

#### MMN

MMN is a negative ERP elicited by the presentation of a “deviant” rare sound in a repetitive sequence of “standard” tones that generally occurs after 150–250 ms upon the presentation of the deviant stimulus (173–176). The deviant stimulus in the auditory modality can differ from the standard one in terms of duration (dMMN) or pitch (pMMN) (177). Currently, a deficit in MMN elicitation is one of the most robust and replicable findings in schizophrenia and it has been related to cognitive dysfunctions, as well as functional impairment in people suffering from this disorder (178–185). Interestingly, the impairment in MMN has been reported already in the early stages of psychotic disorders (63, 65, 186, 187). The reproducibility of dMMN deficit for at-risk and early stages of schizophrenia is greater than that of pMMN, with the latter emerging more robustly only during the chronic stage of schizophrenia. Thus, dMMN can be a more sensitive marker than pMMN, in the context of early psychosis (30, 65).

##### dMMN

Discrepant findings have been reported with the regard to the dMMN amplitude. In particular, some studies found a reduction of dMMN amplitude in FEP (66, 151, 188–191) and FES subjects (65, 95, 181, 182, 190, 192–196) as compared to HCs, while other studies did not find any abnormality in dMMN amplitude in FEP and FES subjects (183, 197–200). Furthermore, a study, involving ML to distinguish FES from HCs with dMMN measures in addition to other neuroimaging and clinical evaluations, highlighted that this EEG-index did not contribute significantly to the discriminant ML-model (158).

A reduction in dMMN amplitude has been reported also in HR subjects (66, 151, 191, 192, 194, 196, 201–204). In particular, it seems that abnormalities in dMMN amplitude might predict the onset of psychosis since they were present in HR-T and not in HR-NT subjects (181, 196, 201–203, 205, 206). However, some studies failed to find differences between HR subjects and HCs in the dMMN amplitude (180, 181, 205, 207).

Most of the studies did not find significant differences between HCs, FEP (188, 189), FES (193, 194, 198, 201, 206), and HR subjects (180, 194, 201, 204, 206, 207) in dMMN latency, with the exception of one study that found delayed latency in dMMN peak in FEP subjects (196), as compared to HCs.

##### pMMN

Findings concerning pMMN amplitude are also inconsistent. Specifically, several studies reported that FES subjects, as compared to HCs, showed a significant reduction of pMMN amplitude (164, 190, 198, 208); a multimodal-longitudinal study showed that the deficit became evident approximately after 1.5 years from illness onset and was correlated with a reduction in Heschl's gyrus volume (198). Other studies, however, did not confirm the above-mentioned results (65, 164, 183, 192, 194, 197, 199, 200, 209–211). As for the dMMN, also the pMMN amplitude did not contribute to the ML model created to differentiate FES subjects from HCs (158).

Most studies reported no abnormalities in pMMN amplitude in HR subjects (both HR-T and HR-NT) (180, 192, 194, 205, 207, 209), while only two studies reported a pMMN amplitude reduction in HR subjects (203, 204). All of the studies that considered pMMN latency did not find significant differences between HCs and FEP (209) or FES (194) or HR subjects (194, 204, 207, 209).

#### P300

The P300 is an ERP positive deflection that appears after the onset of rare deviant “target” stimuli embedded in a sequence of frequent “standard” stimuli (212). Previous reviews have showed impairments of this EEG index in at-risk, prodromal, early, and chronic phases of schizophrenia (33, 63, 67).

P300 is not a unitary phenomenon, but it is composed of 2 functionally different subcomponents: an early component, the P3a, which usually peaks within a time window of 130–275 ms after the stimulus onset, that reflects an involuntary shift in attention toward a deviant stimulus; a later component, the P3b, observed within a time window of 275–600 ms after the stimulus, that reflects a conscious and controlled attentive process toward a stimulus and its task-relevance (60). Some studies explicitly differentiated P3a and P3b, analyzing one or both subcomponents in the same sample, while others referred more generally to P300 recordings. In most of studies which referred to a general P300, P300 was considered as the most positive deflection within a time window of 250–600 ms after the stimulus, thus referring mainly to the P3b component.

In order to record and characterize the P300, most of the studies addressing impairments in at-risk and early stages of schizophrenia have employed visual and auditory tasks. In particular, only three studies used a visual paradigm (161, 165, 170) and found a reduced amplitude (161, 165, 170) and prolonged latency (161, 170) of P300 in FES (161, 165) and HR (161, 165, 170), as compared to HCs. The two following subsections will include results of those studies that recorded P3a and P3b components all during auditory paradigms.

##### P3a

In FEP and FES subjects different studies have highlighted a decrease of the P3a amplitude compared to HCs (163, 188, 189, 194, 196, 200, 209, 210, 213). On the other side, some studies failed to detect differences in P3a amplitude between HCs and FEP (190, 195). Interestingly, in one study, deficits in P3a were not present at the baseline, but emerged when FEP subjects were evaluated at 12 and 24 months follow-up visits (166).

The decrease in P3a amplitude has been reported also in several studies including HR subjects, as compared to HCs (68, 128, 163, 194, 196, 209, 214), while two studies did not find any difference in P3a amplitude between HR and control groups (206, 207). One study found P3a amplitude reduction in HR-T compared to HR-NT (196), while two studies did not find any difference between these two groups (68, 214).

Regarding the P3a latency just one study of those mentioned above highlighted a delayed P3a latency in FES subjects (213), while all other studies did not identify any alteration in P3a latency, in FEP, FES and HR subjects (68, 163, 188, 189, 196, 206, 207, 209, 213–215).

##### P3b

In FEP and FES subjects, as compared to HCs, different studies have highlighted a decrease of the P3b amplitude (67, 163, 164, 172, 199, 210, 213, 215–223). One study demonstrated that deficits in P3b, as observed for P3a, were not present at baseline, but emerged when FEP subjects were evaluated at 12- and 24-month follow-up visits (166). Furthermore, using LORETA, it was shown that the reduction of P3b amplitude was mainly driven by dysfunctions in the left temporal regions (222), while a multimodal study, using MRI and EEG, has shown that P3b reduction was specifically associated with left superior temporal gyrus gray matter volume reduction (224). Only one study did not detect any alteration of the P3b amplitude in FEP subjects, compared to HCs (167).

Different studies have reported how the decrease in P3b amplitude is present since the at-risk phases of the illness (68, 163, 168, 169, 171, 214, 225–227). Interestingly, one study highlighted a step-wise decline in P3b amplitude throughout illness course, characterized by a progressive decrease of P3b in subjects at at-risk, early and chronic stages of SCZ (P3b amplitude in HCs>HR>FEP> chronic SCZ) (226). However, other studies found that P3b amplitude was as impaired in HR as in subjects at more advanced stages of illness (163, 214, 227). Several studies showed P3b amplitude decrease in HR-T compared to HR-NT and HCs (68, 171, 214), while just one study did not detect any difference in P3b amplitude between converters and not converters (225). A study divided the HR sample in HR-T and HR-NT and the authors performed a further subdivision for the latter group into remitted and non-remitted. It was observed that HR-T subjects and HR-NT who continued to present attenuated symptoms showed reduced P300 compared to HR-NT who remitted and HCs (228).

With regard to the P3b latency, two studies reported an increase of this EEG measure in FEP (215, 229), while other studies did not detect any abnormalities of this feature in FEP, FES (162, 163, 165, 167, 172, 213, 216–219, 221, 222, 224, 227, 230–232), and HR subjects (68, 163, 165, 168, 169, 227, 228), as compared to HCs.

#### N400

N400 is a negative-going deflection that peaks around 400 ms post-stimulus onset and it is typically maximal over centro-parietal electrode sites. The N400 is part of the normal brain response to words and other meaningful stimuli, including visual and auditory words, sign-language, pictures, faces, environmental sounds, and smells (233). Several studies have revealed that subjects with chronic schizophrenia presents abnormalities in this ERPs values, with some results suggesting that N400 semantic priming deficits may reflect an underlying neurophysiological mechanism of delusions (234).

However, to date, very few articles have addressed this EEG-index in early stages of schizophrenia. In one study, N400 presented a reduced amplitude and a prolonged latency in FES, as compared to HCs (235). The reduced N400 amplitude has also been found in HR subjects, as compared to HCs (128), and was associated with neurocognitive impairment (236).

## Discussion

EEG recordings provide *in-vivo* access to neuronal activity and each EEG index can reflect distinct sensory and cognitive processes. This review illustrates how multiple EEG-based indices result already altered during at-risk and early stages of schizophrenia, supporting the hypothesis that cerebral networks dysfunctions appear early in the course of the disorder (25, 237, 238). However, although a large number of studies have highlighted differences between FEP or HR subjects and HCs on EEG variables, only few of these showed homogeneous and consistent results.

### EEG Frequency Bands

Studies on frequency bands have reported several abnormalities across all five bands in at-risk and early stages of schizophrenia.

Within delta band, an increase in resting-state activity in both at-risk and first-episode subjects compared to HCs has often been reported (75–79). Studies have suggested that dysfunctions in this band might arise from changes in dopamine synthesis levels in the fronto-striatal-thalamic loops, which are detectable already at the onset of psychotic disorders and result associated to the severity of prodromal psychotic symptoms (29, 239). Furthermore, this EEG index has also been successfully used to predict the trajectory of negative symptoms and functioning in FES subjects (76).

Studies on theta and alpha bands reported mixed results (73, 75–77, 87, 88, 100). However, the activity of these two bands during a task performance revealed alterations in both FEP and HR samples in almost all of the studies considered. The association of these bands to clinical symptoms (88, 91, 94) and cognitive domains (91, 92, 94, 95) suggests that abnormalities in neuronal oscillations in these frequency bands could contribute to the clinical presentation in early disease stages.

For the beta band, only few studies (78, 88, 89) reported alterations, while the majority did not find significant abnormalities in early and at-risk subjects (73–77, 109). The same is true for studies investigating stimuli-related activity, coherence, and connectivity measures in this band.

Finally, for the gamma band, abnormalities of the resting-state power, evoked power, synchrony, coherence, and connectivity were observed in the early stages of schizophrenia (62). Therefore, considering the role of gamma oscillations in the cognitive processes (110, 111), the widespread cognitive deficits observed already at early stages of schizophrenia (17, 240, 241) might be connected to abnormalities in the gamma activity across cerebral networks.

Considering these results on the whole, it is important to underline that the diversity of the EEG paradigms and analysis methods employed in the studies, do not allow drawing solid conclusions.

### Microstates

Abnormalities in the characteristics of MS, such as their mean duration and the presence of abnormal patterns in the MS syntax, suggest that sustainment of neuronal activity in interconnected cerebral regions is impaired already at early stages of the disorder (25, 54, 134–136).

Furthermore, in a recent study, which included FEP, HR-T, HR-NT, and HCs, the authors discussed how abnormalities of different microstate parameters might be linked to different aspects of the illness (242). In particular, abnormalities of MS-A in FEP and HR subjects could represent an unspecific state biomarker of general psychopathology; abnormalities of MS-B in FEP may represent a state biomarker specific to psychotic illness progression; and finally, abnormalities of MS-D in HR-T (and not HR-NT) might represent a biomarker of future transition of HR subjects (242). However, to date, very few articles have investigated the microstates in HR and FEP subjects, revealing a research gap opportunity for future EEG studies.

### ERPs

The studies included in the current review showed that in FEP and HR subjects compared to HCs, all considered ERPs presented abnormalities, generally manifested as a reduction in their amplitude. These deficits were detected both for ERPs linked to the basic levels of sensory processing, such as P50 and N100, and for indices related to higher levels of cognitive functions, such as MMN and P3b (63). For instance, the results reported for P50, support previous evidence that sensory gating is already impaired in at-risk and early stages of schizophrenia and may reflect a diminished capacity to filter repetitive sensory signals (44). Conversely, impairments in N100 seem to be more consistent in FEP rather than HR subjects, suggesting that this type of deficit might emerge only at a morbid stage of the disorder. The relationship of this ERP abnormalities with primary negative symptoms (160, 243), which might be present only in a subgroup of subjects with schizophrenia, might partially explain the heterogeneity of the results. One general limitation of the studies addressing at-risk and prodromal stages is the poor characterization of negative symptoms and cognitive deficits, which are not included in diagnostic criteria though they might be predictive of poor clinical and functional outcome and conversion to schizophrenia (18). Recent data have demonstrated that early auditory processing deficits, as assessed either by neuropsychological tasks or MMN (244, 245), are present only in a subgroup of subjects with schizophrenia. In at-risk subjects the same deficits are not always found (244), indicating that either cognitive and neurophysiological deficits develop only from the onset of the morbid phase or that individuals with premorbid deficits are not being captured by current criteria which do not consider core aspects of schizophrenia-spectrum disorders, such as negative symptoms.

Amongst the ERPs indices, MMN and P300 showed the highest rate of abnormal values in the studies included in the present review. Articles focusing on MMN have shown that reductions in the amplitude of dMMN and pMMN seem to be different depending on the phase of the disorder. Reductions in dMMN, in fact, precedes the one in pMMN, which is almost never observed in HR subjects and only few times in FEP subjects (64). Thus, dMMN can be a more sensitive marker than pMMN in the context of prodromal and early psychosis (30, 65, 95, 185, 186). Alternatively, pMMN reductions could reflect illness chronicity and could be used to monitor treatment efficacy and disease progression (64, 198). In addition to MMN results, also P300 might be an index used as a robust and effective biomarker for transition to psychosis in HR and for prognosis of the disorder in FEP subjects (67, 68, 171, 214, 226).

Finally, the low number of studies measuring N400 in FEP and HR prevents the formulation of any inference on this component.

### Limits of EEG Indices in the Early Stages of Schizophrenia

The integration of the results reported in the current systematic review is compromised by three major limitations: the use of different diagnostic criteria and assessment scales, the heterogeneity of EEG paradigms and analysis methods used in the included studies and, finally, the intra and inter-subjects' variability of EEG recordings.

Firstly, both for at-risk and early phases of schizophrenia, inclusion criteria were based on different operational definitions. For instance, for at-risk or prodromal phases, ARMS, UHR, and CHR operational criteria have been used in different studies. In addition, the occurrence of a first episode was defined as the first contact at a clinical setting, or the duration of antipsychotic medication use or the duration of psychotic symptoms (26).

Secondly, there was a huge heterogeneity in experimental paradigms, types of EEG indices considered and the definition of their characteristics such as band ranges cut-off points and the employed analysis methods. This was mostly evident in frequency band rather than in ERP studies.

Finally, EEG studies are generally characterized by a high variability within and between subjects, which might not always be indicative of any pathological status, making difficult a generalization of the reports. In fact, EEG data recordings might be influenced by normal temporal fluctuations in subject physiology, as well as caffeine and nicotine intake, skull conductivity, several medical conditions, and medication (246, 247).

### Current and Future Perspective on the Employment of EEG-Indices in Clinical Settings

The application of EEG indices into clinical settings to predict the likelihood of conversion to psychosis from a high-risk state or the onset and the progression of the illness is still under scrutiny. Due to the high variance of the results considered in the present review, none of these EEG indices can be regarded as a flawless marker of at-risk or early stages of schizophrenia, and no clinical translation has yet been envisaged for any of them (62, 64, 68). For instance, alterations in EEG-based measures such as N100, MMN or P300 are indicators of deficits in early sensory processing. However, the literature reported that these impairments are dichotomously distributed among schizophrenia subjects during their early and chronic stages of illness, so they are not found in all affected patients. For this reason, also in subjects at-risk of schizophrenia, deficits in early sensory processes are not always reported, leading to discrepant results. Furthermore, it should be considered that the presence of sensory processing deficits is not included among ARMS criteria, which might imply that some subjects can be missed using current diagnostic systems (244, 245).

Another consideration that should be made, is that most of the studies included in the review rely on comparisons of HR and FEP subjects to HCs on only one EEG index, while only few analyzed multiple EEG indices simultaneously (76, 79, 163, 248, 249). The latter approach could be more effective in predicting clinical and functional outcome both in early and prodromal phases of schizophrenia or to characterize the neurobiological alterations in initial phases. For instance, Renaldi et al. (76) used a multiple regression analysis with delta, theta, alpha, and beta spectral power with the aim to predict symptomatic and functional improvement in FES subjects. Furthermore, a study investigated the utilization of N100, P3a, and P3b amplitudes to discriminate HR and FES subjects from HCs, showing that only the first two EEG-indices were significant predictors of the diagnosis (163). Some authors analyzed changes in multiple frequency band indices, using a longitudinal design, to identify which could predict conversion to psychosis in HR subjects (79, 248, 249). In one of these studies, delta, theta and alpha activity contributed to a predictive model of conversion (79). Furthermore, another study (249) showed that a regression model using delta, theta, and beta activity, combined with clinical data, was able to predict with an accuracy of over 80% the transition to psychosis in HR subjects. Conversely, one study reported that absolute power values of frontal alpha, beta, and delta activity were not associated to transition to psychosis (248).

In the last decades, machine learning technique became popular to overcome the limits of univariate analyses, which require a preselection of variables to be used in prediction. This promising approach is based on the use of multiple variables (e.g., electrophysiological, genetic, or clinical data) and the algorithm enables general hypotheses and previsions (e.g., discrimination between patients and controls, prediction of the response to treatment or clinical course). This approach has been used in different imaging studies, with the aim to predict clinical information at individual level (250–252). However, in studies with subjects at early and prodromal stages of schizophrenia, only few research groups have employed this promising method with EEG data. Among these, one study utilized the machine learning algorithm, which incorporated measures of current-source density (CSD) and LORETA synchronization indices of beta and gamma oscillations, in order to predict HR subjects who would develop psychosis in a 3-years follow-up (253). Furthermore, another study found that EEG-measures, such as P50 and MMN, could identify the presence of two distinct subgroups within a FES sample, which could potentially assist clinicians in treatment design based on the individual neurobiological differences (254). In a recent longitudinal study (255), machine learning was used to discriminate two subgroups of FEP subjects, according to changes in dMMN amplitude, revealing that subjects with improvement of dMMN had better clinical, cognitive, and functional follow-up outcomes than those with worsening of dMMN (255). However, another study did not find a significant contribution of electrophysiological indices, such as P50 and MMN, for the discrimination between FES and HCs (158).

Besides the multivariate approach, it could be very useful to use multimodal analyses in which, for instance, simultaneous EEG-MRI is recorded in order to achieve a good spatial-temporal resolution. However, few of the included studies have been conducted using a multimodal approach, analyzing and correlating MRI and EEG abnormalities in these groups of subjects (124, 198, 224, 256).

Finally, given that progressive changes in EEG measures are associated with transition to psychosis and disease course, more studies with a longitudinal design (76, 79, 121, 255, 256) are needed.

## Conclusions

The current systematic review advocates the conduction of further studies on EEG indices that could support clinicians in their decision-making process in the early stage of the disorder. In order to draw reliable conclusions from the combination of the various studies, standardized subjects' inclusion criteria, electrophysiological protocols, and analysis methods should be adopted.

Studies should include multiple EEG indices, integrate them with other clinical variables and apply multivariate approaches, such as machine learning algorithms, in order to provide a reliable tool in the diagnosis and prognosis of early and prodromal stages of schizophrenia.

## Data Availability Statement

The original contributions presented in the study are included in the article/Supplementary Material, further inquiries can be directed to the corresponding author.

## Author Contributions

AP, GG, and AM contributed to the conceptualization and supervision of the manuscript. AP, GG, and FB contributed to the establishment of the methodology and the literature research. All authors contributed to writing, critically revising, and editing the content of the manuscript and approved the final manuscript for submission to Frontiers in Psychiatry.

## Conflict of Interest

General sources of potential conflict of interest, considered unrelated to this work include the following: AM received honoraria, advisory board or consulting fees from the following companies: Amgen Dompé, Angelini-Acraf, Astra Zeneca, Bristol-Myers Squibb, Gedeon Richter Bulgaria, Innova-Pharma, Janssen Pharmaceuticals, Lundbeck, Otsuka, Pfizer and Pierre Fabre. The remaining authors declare that the research was conducted in the absence of any commercial or financial relationships that could be construed as a potential conflict of interest.
