# Supplementary material for: EEG-Based Measures in At-Risk Mental State and Early Stages of Schizophrenia: A Systematic Review
Source: Front Psychiatry. 2021 May 4;12:653642. doi: 10.3389/fpsyt.2021.653642 (PMC8129021; doi:10.3389/fpsyt.2021.653642)
Supplement: Supplementary file 1 [file Table_1.docx]

**Table S1. Frequency bands studies**

| **STUDY** | **CONDITION/ BAND/ MEASURE** | **N** | **DIAGNOSTIC INSTRUMENT** | **RESULTS** |
| --- | --- | --- | --- | --- |
| Alexander et al., 2009 (82) | **Task**  Delta  Spatio-temporal analysis of waves propagation | FES = 46  HCs = 94 | DSM-IV | **Occurrence of anterior-to-posterior delta waves**  FES < HCs (anterior-to-posterior waves) |
| Andreou et al., 2015 (91) | **Resting-state**  Gamma  Power  Functional connectivity | FES = 22  HCs = 22 | MINI | **Power**  No differences  **Functional Connectivity**  FES > HCs |
| Andreou et al., 2015 (92) | **Resting-state**  Theta  Functional connectivity | CHR=28  FES = 19  HCs =23 | SPI-A  GAF  SIPS  SOPS  MINI | **Functional Connectivity**  UHR = HCs  FES > HCs |
| Begré et al., 2003 (73) | **Resting-state** Delta,theta,alpha,beta Functional connectivity (LORETA)  Power | FES = 7  HCs = 7 | DSM-IV | **Power**  No differences  **Connectivity**  Alpha  FES < HCs (inferior  parietal lobule)  FES > HCs (superior frontal gyrus)  Delta, theta, beta  No differences |
| Boudewyn and Carter, 2018 (94) | **Task**  Alpha, theta  Response-related activity | FES = 75  HCs = 57 | SCID (DSM-IV)  K-SADS-PL | **Activity after errors**  Theta  FEP < HCs  Alpha  FEP > HCs |
| Clementz et al., 1994 (75) | **Resting-state**  Delta, theta, alpha, beta  Power | FES = 50  HCs = 155 | Clinical assessment and “Present State Examination” | **Power**  Delta  FES > HCs  Theta  FES > HCs  Alpha  FES < HCs  Beta  No differences |
| Flynn et al., 2008 (116) | **Task**  Gamma  Phase synchrony | FEP = 55  HCs = 110 | SCID (DSM-IV) | **Phase synchrony**  Gamma  FEP > HCs |
| Garakh et al., 2015 (88) | **Resting-state** **and** **Task**  Delta, theta, alpha, beta, gamma  Power | FES = 32  HCs = 40 | ICD-10 | **Resting-state-Power**  Delta  No differences  Theta  FES > HCs  Alpha  FES < HCs  Beta  FES > HCs  **Task - Power**  Theta, Beta, Gamma,  FES < HCs  (midline theta, left anterior beta, and anterior  gamma power)  Alpha  No differences |
| Harris et al., 2006 (77) | **Resting-state**  Delta, theta, alpha, beta  Peak Frequency  Power | FES = 40  HCs = 40 | DSM-IV | **Peak frequency**  Delta  FES < HCs  Theta, alpha, beta  No differences  **Power**  Delta  FES > HCs  Theta, alpha, beta  No differences |
| Jhung et al., 2013 (96) | **Task**  Delta, theta, alpha, beta, gamma  Functional connectivity (within “small-world networks”) | UHR = 13  FES = 13  HCs = 13 | SIPS  SCID (DSM-IV) | **Small world index**  Theta  UHR = HCs  FES < HCs  Delta, alpha, beta, gamma  No differences  **Clustering coefficient**  Alpha  UHR > HCs  FES = HCs  Delta, theta, beta, gamma  No differences  **Path length**  No differences |
| Kayser et al., 2014 (103) | **Task**  Alpha  ERD | CHR = 22  HCs = 20 | SIPS  SOPS | **ERD**  CHR < HCs |
| Koenig et al., 2001 (74) | **Resting-state**  Delta, theta, alpha, beta  GFS | FES = 28  HCs = 28 | DSM-III-R | **GFS**  Theta  FES < HCs  Delta, alpha, beta  No differences |
| Krukow et al., 2018 (93) | **Resting-state**  Theta, alpha, gamma  PLI | FES = 32  HCs = 35 | SCID (DSM-V) | **PLI**  Theta  FES > HCs  Alpha  FES < HCs  Gamma  FES < HCs |
| Krukow et al., 2020 (80) | **Resting-state**  Delta, theta, alpha, beta, gamma  Synchronization (LORETA) | FES = 34  HCs = 30 | SCID (DSM-V) | **Synchronization**  Delta  FES < HCs (lateral parts of the parietal, frontal and temporal cortex)  Theta  FES > HCs (posterior cingulate cortex, cuneus, precuneus)  Alpha, beta, gamma  FES < HCs (generalized) |
| Lee et al., 2020 (90) | **Task**  Theta phase-gamma amplitude coupling | FEP = 59  HCs =50 | SCID (DSM-IV) | **Theta phase-gamma amplitude coupling**  FEP > HCs (posterior cingulate cortex) |
| Leicht et al., 2015 (122) | **Task**  Gamma  Power  PLF | FES = 21  HCs = 21 | MINI | **Evoked power**  FES < HCs  **PLF**  FES < HCs |
| Leicht et al., 2016 (124) | **Task**  Gamma  Power  Peak frequency | CHR = 27  HCs =26 | SPI-A  GAF  SIPS  SOPS | **Power**  CHR < HCs  **Peak frequency**  CHR < HCs |
| Lepock et al., 2019 (128) | **Task**  Gamma  ASSR power  PLF | CHR = 36  HCs = 22 | SIPS | **ASSR power**  No differences  **PLF**  No differences |
| Liu et al., 2019 (102) | **Resting-state**  Alpha  PLI  Global network analysis | UHR = 21  FES = 28  HCs = 28 | SIPS  DSM-IV | **PLI**  UHR > HCs  FES > HCs  **Global efficiency**  UHR > HCs  FES > HCs  **Local efficiency**  UHR > HCs  FES > HCs  **Path length**  UHR < HCs  FES < HCs |
| Minzenber et al., 2010 (115) | **Task**  Gamma  Power | FES = 53  HCs = 29 | SCID (DSM-IV)  K-SADS-PL | **Power**  FES < HCs |
| Missonnier et al., 2017 (114) | **Task**  Gamma  Band variability  Signal complexity | FES =15  HCs =18 | DSM-IV | **Band variability**  FES > HCs  **Signal Complexity**  FES > HCs |
| Missonnier et al., 2020 (81) | **Task**  Gamma, delta  Event-related activity | FEP = 15  HCs = 18 | DSM-IV | **Synchronicity**  Gamma  FEP < HCs  **Period oscillation**  Delta  FEP < HCs |
| Murphy and Öngür, 2019 (100) | **Resting-state**  Alpha  Peak frequency  Power  (LORETA) | FEP = 22  HCs = 22 | SCI-PANSS  SCID (DSM-V) | **Peak frequency**  FEP < HCs (frontal, parietal, temporal, and occipital  cortex)  **Power**  No differences |
| Oribe et al., 2019 (118) | **Task**  Gamma (BL and 1 year FU)  PLF amplitude  Peak frequency  Evoked power | CHR = 18  FES = 18  HCs = 40 | SOPS  SCID (DSM-IV) | *BL*  **PLF amplitude;Peak frequency**  No differences  **Evoked power**  No differences.  *FU (1 year)*  **PLF amplitude**  CHR = HCs  FES < HCs  **Peak frequency**  CHR = HCs  FES > HCs  **Evoked power**  No differences |
| Pascual-Marqui et al., 1999 (78) | **Resting-state**  Delta, theta, alpha, beta  Power (LORETA) | FES = 9  HCs = 36 | DSM-IV | Delta  FES > HCs (prefrontal areas)  Theta  FES < HCs (anterior regions)  Alpha  FES < HCs (temporal-parietal areas)  Beta  FES > HCs (right  parietal area) |
| Perez et al., 2013 (123) | **Task**  Gamma  Power | CHR = 43  HCs = 42 | SIPS | **Power**  CHR < HCs |
| Ramyead et al., 2016  (89) | **Resting-state**  CSD (LORETA)  LPS | FEP = 31  HCs =29 | BSIP | **CSD**  Theta  FEP < HCs (left anterior cingulate)  Alpha  FEP < HCs (left middle frontal gyrus)  Beta  FEP > HCs (superior frontal gyrus)  Gamma  FEP>HCs (left medial frontal gyrus)  **LPS**  Diffused alterations in FEP, revealing deficits in synchronization of long-range activity, especially in the beta band |
| Ramyead et al., 2015 (113) | **Resting-state**  Theta, alpha, beta, gamma  CSD (LORETA)  LPS | ARMS = 63  (ARMS-NT = 40;  ARMS-T = 23)  HCs = 29 | BSIP | **CSD**  Delta  ARMS-T activity  distributed  in frontal and parieto-occipital areas, while  in HCs and ARMS-NT activity was more localized  in frontal cortex  Theta, alpha, beta  No differences  Gamma  ARMS-T > ARMS-NT/HCs (in the medial prefrontal cortex)  **LPS**  ARMS-T LPS  of beta oscillations decreased more over Euclidian distance compared to ARMS-NT and HCs |
| Ranlund et al., 2014 (87) | **Resting state**  Delta, theta, alpha, beta  Power | ARMS = 33  FEP = 46  SCZ = 48  HCs= 107 | CAARMS  SCID (DSM-IV) | **Power**  Delta  SCZ > HCs  FEP = HCs  ARMS = HCs  Theta  SCZ > HCs  FEP = HCs  ARMS = HCs  Alpha  No differences  Beta  No differences |
| Reilly et al., 2018 **(Systematic Review)** (62) | **Review** on Gamma band activity | 45 Studies included (44 with FEP, FES and 16 with CHR) | N.A. | N.A. |
| Renaldi et al., 2019 (76) | **Resting-state**  Delta, theta, alpha, beta  Power | FEP = 24  HCs = 24 | SCID (DSM-IV) | **Power**  Delta  FEP > HCs  Theta, alpha, beta  No differences |
| Slewa-Younan et al., 2004 (119) | **Task**  Gamma  Phase synchrony | FES = 24  SCZ = 40  HCs = 24 | DSM-IV  ICD-10 | **Synchrony Magnitude**  FES < HCs  SCZ < HCs  **Synchrony Latency**  FES > HCs (frontal regions) |
| Spencer et al., 2008 (125) | **Task**  Gamma  ASSRs | FES = 16  HCs = 33 | SCID (DSM-IV) | **Phase locking**  FES < HCs (for 30 and 40 Hz; not for 20 Hz)  **Evoked power**  FES < HCs (for 30 and 40 Hz; not for 20 Hz) |
| Symond et al., 2005 (120) | **Task**  Gamma 1, Gamma 2  Global synchrony  Latency | FES = 40  HCs = 40 | DSM-IV  ICD-10 | Gamma 1  **Global synchrony**  FES < HCs (anterior regions)  **Latency**  FES > HCs  Gamma 2  No differences |
| Tada et al., 2016 (126) | **Task**  Gamma  ITC  ERSP | UHR= 15  FES= 13  HCs=21 | SIPS  DSM-IV | **ITC**  UHR < HCs (late phases)  FES < HCs (early and late phases)  **ERSP**  UHR < HCs (late phases)  FES < HCs (late phases) |
| Tikka et al., 2014 (112) | **Resting-state**  Gamma  Power | FES = 37  HCs = 30 | ICD-10 | **Power**  FES > HCs |
| van Tricht et al., 2014 (79) | **Resting-state**  Delta, theta, beta  Power  Alpha  Power  Peak frequency | CHR = 113 (CHR-T=22; CHR-NT=91)  HCs = 54 | SIPS  GAF | **Power**  Delta  CHR-T > CHR-NT > HCs  Theta  CHR-T > CHR-NT > HCs  Alpha, beta  No differences  **Peak frequency**  Alpha  CHR-T > CHR-NT > HCs |
| Wang et al., 2018 (127) | **Task**  Gamma  ITC  ERSP | FES= 33  HCs= 28 | SCID (DSM-IV) | **ITC**  FES < HCs  **ERSP**  FES < HCs |
| Williams et al., 2009a (121) | **Task**  Gamma  Synchrony | FES = 35  HCs = 35 | SCID (DSM-IV) | **Synchrony**  FES < HCs |
| Williams et al., 2009b (117) | **Task**  Gamma  Synchrony | FES= 28  HCs= 28 | SCID (DSM-IV) | **Synchrony**  FES > HCs (temporal and temporo-frontal regions) |
| Xiong et al., 2019 (95) | **Task**  Theta  Power | FES = 40  SCZ= 40  HCs = 40 | SCID (DSM-IV) | **Power**  FES < HCs  SCZ < HCs |
| Yeragani et al., 2006 (109) | **Resting-state**  Beta, gamma  Power  EEG coherence | FES = 8  HCs = 8 | SCID (DSM-IV) | **Power**  Beta, gamma  No differences  **EEG Coherence**  Beta, gamma  FES < HCs |
| Zaytseva et al., 2018 (101) | **Resting-state** and **task**  Delta, theta, alpha, beta, gamma  EEG coherence | FES = 32  HCs = 40 | ICD-10 | **Resting state -Coherence**  Alpha  FES < HCs  Beta  FES < HCs  Gamma  Mixed results depending on the electrode site.  **Task-Coherence**  Alpha  FES < HCs  Beta  FES < HCs  Gamma  Mixed results depending on the electrode site. |
| Zhao et al., 2018 (83) | Delta, theta, alpha, beta  EEG signal complexity (path length) | FES = 14  HCs = 14 | SCID (DSM-IV) | **EEG complexity**  Theta  FES > HCs  Delta, Alpha, Beta  No differences |

At Risk Mental State (ARMS); ARMS who did transition (ARMS-T); ARMS who did not transition (ARMS-NT); Auditory steady-state EEG responses (ASSRs); Basel Screening Instrument for Psychosis (BSIP); Baseline assessment (BL) Comprehensive Assessment of At Risk Mental States (CAARMS); Clinical High Risk (CHR); CHR who did transition (CHR-T); CHR who did not transition (CHR-NT); Current Source Density (CSD); Diagnostic and Statistical Manual of Mental Disorders (DSM); Event-Related Desynchronization (ERD); Event-related spectral perturbation (ERSP); First-Episode Psychosis (FEP); First-Episode Schizophrenia (FES); Follow-up assessment (FU); Global Assessment of Functioning scale (GAF); Global Field Synchronization (GFS); Healthy Control (HCs); International Statistical Classification of Diseases (ICD); Intertrial coherence (ITC); Kiddie-SADS-Present and Lifetime Version (K-SADS-PL); Low Resolution Electromagnetic Tomography (LORETA); Lagged Phase Synchronization (LPS); Mini-International Neuropsychiatric Interview (MINI); Positive and Negative Syndrome Scale (PANSS); Phase-Locking Factor (PLF); Phase Lag Index (PLI); Structured Clinical Interview for the Diagnostic and Statistical Manual of Mental Disorders (SCID); Structured Clinical Interview for the Positive and Negative Symptom Scale (SCI-PANSS); Chronic Schizophrenia (SCZ); Structured Interview of Psychosis-risk Syndromes (SIPS); Schizophrenia Proneness Instrument, Adult Version (SPI-A); Scale of Psychosis-Risk Symptoms (SOPS); Ultra High Risk (UHR).
