# Supplementary material for: EEG-Based Measures in At-Risk Mental State and Early Stages of Schizophrenia: A Systematic Review
Source: Front Psychiatry. 2021 May 4;12:653642. doi: 10.3389/fpsyt.2021.653642 (PMC8129021; doi:10.3389/fpsyt.2021.653642)
Supplement: Supplementary file 2 [file Table_2.docx]

**Table S2. Microstates studies**

| **STUDY**  (first author et al., year) | **MEASURE** | **N** | **DIAGNOSTIC INSTRUMENT** | **RESULTS** |
| --- | --- | --- | --- | --- |
| Andreou et al., 2014  (136) | Characteristics of MS-A, MS-B, MS-C, MS-D | HR = 18  SCZ = 18  HCs = 22 | GNRS  MINI | **MS-A**  Higher coverage and occurrence in HR than SCZ and HCs  **MS-B**  Covered more time in SCZ compared to both HR and HCs.  **MS-C**  No significant differences  **MS-D**  No significant differences  Aberrant spatial configuration in SCZ, and to a lesser extent also in HR, compared to HCs |
| Begré and Koenig, 2008 **(Review)** (25) | **Review** on disconnectivity measures | 3 relevant studies on microstates | N.A. | N.A. |
| Koenig et al., 1999 (54) | Characteristics of MS-A, MS-B, MS-C, MS-D | FEP = 9  HCs = 8 | DSM-IV | **MS-A**  No significant differences  **MS-B**  No significant differences  **MS-C**  No significant differences  **MS-D**  Reduced duration in FEP |
| Lehmann et al., 2005  (134) | Characteristics of MS-A, MS-B, MS-C, MS-D | FES = 27  HCs = 27 | DSM-III-R DSM-IV  ICD-9 | **MS-A**  In FES occurred more frequently and covered more time  **MS-B**  In FES was shortened, covered less time  **MS-C**  In FES occurred more frequently  **MS-D**  In FES was shortened |
| Tomescu et al., 2014 (135) | Characteristics of MS-A, MS-B, MS-C, MS-D | Adolescents with 22q11DS = 30  HCs = 28 | SIPS | **MS-A**  No significant differences  **MS-B**  No significant differences  **MS-C**  Increased presence in the 22q11DS adolescents with respect to controls  **MS-D**  No significant differences |

Diagnostic and Statistical Manual of Mental Disorders (DSM); First-Episode Psychosis (FEP); First-Episode Schizophrenia (FES); Criteria of the Early Detection and Intervention program of the German Research Network on Schizophrenia (GNRS); Healthy Controls (HCs); High Risk (HR); International Statistical Classification of Diseases (ICD); Mini International Neuropsychiatric Interview (MINI); Microstate A (MS-A); Microstate B (MS-B); Microstate C (MS-C); Microstate D (MS-D); Structured Interview of Psychosis-risk Syndromes (SIPS); Chronic Schizophrenia (SCZ).
