# Supplementary material for: EEG-Based Measures in At-Risk Mental State and Early Stages of Schizophrenia: A Systematic Review
Source: Front Psychiatry. 2021 May 4;12:653642. doi: 10.3389/fpsyt.2021.653642 (PMC8129021; doi:10.3389/fpsyt.2021.653642)
Supplement: Supplementary file 3 [file Table_3.docx]

**Table S3. Event-Related Potentials studies**

| **STUDY** | **MEASURE** | **N** | **DIAGNOSTIC INSTRUMENT** | **RESULTS** |
| --- | --- | --- | --- | --- |
| Atagun et al., 2020 **(Meta-analysis)**(44) | Meta-analysis of **P50** difference* and ratio** | Total of 115 Studies with SCZ (including studies with FEP; number of FEP studies not specified) | N.A. | **P50 difference**  FEP > HCs  **P50 Ratio**  FEP > HCs |
| Atkinson et al., 2012  (196) | **dMMN** amplitude **dMMN** latency  **P3a** amplitude  **P3a** latency | UHR = 30 (UHR-T=6, UHR-NT=24)  FEP = 10  HCs = 20 | CAARMS  DSM-IV | **dMMN amplitude**  FEP < HCs  UHR < HCs  UHR-T < UHR-NT **dMMN latency**  FEP > HCs  **P3a amplitude**  UHR < HCs  FEP < HCs  UHR = FEP  UHR-T < UHR-NT  **P3a latency**  No differences |
| Atkinson et al., 2017 (207) | **dMMN** amplitude  **fMMN** amplitude  **dMMN** latency  **fMMN** amplitude  **P3a** amplitude  **P3a** latency | UHR = 80 (  No Follow-up data=19;UHR-T=6, UHR-NT=55)  HCs = 58 | CAARMS | **dMMN/fMMN amplitude**  No differences  **dMMN/fMMN latency**  No differences  **P3a amplitude**  No differences  **P3a latency**  No differences |
| Bodatsch et al., 2011 (205) | **dMMN** amplitude  **pMMN** amplitude | HCs = 67  FES = 33  AR = 62  (AR-C=25; AR-NC=37) | ERIraos  DSM-IV | **dMMN amplitude**  AR = HCs  AR = FES  AR-C < AR-NC  AR-C < HCs  AR-NC = HCs  AR-NC > AR-C  AR-NC > FES  AR-C = FES  **pMMN amplitude**  AR-C = AR-NC |
| Bramon et al., 2008 (168) | **P300** amplitude  **P300** latency  **N100** amplitude  **N100** latency | ARMS = 35  HCs = 57 | CAARMS | **P300 amplitude**  ARMS < HCs  **P300 latency**  No differences  **N100 amplitude**  No differences  **N100 latency**  No differences |
| Brockhaus-Dumke et al. 2005 (180) | **dMMN** amplitude  **pMMN** amplitude **dMMN** latency  **pMMN** latency | PP = 42  SCZ = 31  HCs = 33 | BSABS  DSM-IV | **dMMN amplitude**  SCZ < HCs  PP = HCs  **pMMN amplitude**  No differences  **dMMN latency**  No differences  **pMMN latency**  No differences |
| Brockhaus-Dumke et al., 2008 (143) | **P50** difference*  **P50** ratio**  **N100** difference*  **N100** ratio**  **N100** S1 amplitude  **N100** S2 amplitude  **N100** S1 latency  **N100** S2 latency | Prodromal state = 39 (AR=18; PP=21)  FEP = 46  SCZ = 20  HCs = 46 | BSABS  SPI-A  DSM-IV | **P50 difference**  SCZ > HCs  SCZ > AR  SCZ > PP  SCZ > FES  **P50 ratio**  CHR > HCs  PP > HCs  FES > HCs  SCZ > HCs  **N100 difference**  AR = HCs  SCZ > All  FEP >HCs  PP = HCs  **N100 ratio**  No differences  **N100 S1 amplitude**  AR = HCs  SCZ < All groups  FEP < HCs  PP = HCs  **N100 S2 amplitude**  No differences  **N100 S1 latency**  No differences  **N100 S2 latency**  No differences |
| Brown et al., 2002 (162) | **N100** amplitude  **N100** latency  **P300** amplitude  **P300** latency | FES = 40  SCZ = 40  HCs FES-paired = 40  HCs SCZ-paired = 40 | Composite  International Diagnostic Interview  Or DSM-III-R | **N100 amplitude**  SCZ < HCs SCZ-paired  FES < HCs FES-paired  **P300 amplitude**  SCZ < HCs SCZ-paired  FES < HCs FES-paired  **P300 latency**  No differences  **N100 latency**  No differences |
| Carrion et al., 2015 (204) | **dMMN** amplitude  **pMMN** amplitude **dMMN** latency **pMMN** latency | CHR = 34  HCs = 33 | SIPS | **dMMN**  CHR < HCs  **pMMN**  CHR < HCs  **dMMN latency**  No differences  **pMMN latency**  No differences |
| Chang et al., 2019  (154) | **P50** difference*  **P50** ratio**  **Functional connectivity** (LORETA) | FES = 35  UHR = 30  HCs = 40 | DSM- IV  SIPS-COPS | **P50 difference**  No differences  **P50 ratio**  No differences  **Connectivity**  FES > HCs (right superior frontal gyrus and right insula)  UHR > HCs (paracentral lobule and the middle temporal gyrus) |
| del Re et al., 2015  (163) | **P3a** amplitude  **P3a** latency  **P3b** amplitude  **P3b** latency  **N100** amplitude  **N100** latency | CHR = 21 (0% transition)  FES = 20  HCs = 25 | SIPS  BSABS  COPER  SPI-A  SCID (DSM-IV-TR) | **P3a amplitude**  FES < HCs  CHR < HCs  FES = CHR  **P3b amplitude**  FES < HCs  CHR < HCs  FES = CHR  **N100 amplitude**  FES < HCs  CHR < HCs  FES = CHR  **P3b latency**  No differences  **P3a latency**  No differences  **N100 latency**  No differences |
| Demiralp et al., 2002 (229) | **P3b** amplitude  **P3b** latency | FEP = 12  HCs = 12 | SCID (DSM-II-R) | **P3b amplitude**  FEP < HCs  **P3b latency**  FEP < HCs |
| Devrim-Üçok et al., 2006 (230) | **P3** Novelty amplitude  **P3** Novelty latency  **P3b** amplitude  **P3b** latency | FES = 31  SCZ = 26  HCs younger = 36  HCs older = 35 | SCID (DSM-IV) | **P3b amplitude**  FES < HCs SCZ < HCs  FES = SCZ  **P3 Novelty amplitude**  FES = HCs  SCZ < HCs  **P3b latency**  No differences  **P3 Novelty latency**  No differences |
| Devrim-Uçok et al., 2008a (208) | **pMMN** amplitude | FES = 30 (FES-A = 21; FES-PA = 9)  HCs= 34 | SCID (DSM-IV) | **pMMN**  FES = HCs  FES-A = HCs  FES-PA < HCs  FES-PA < FES-A |
| Devrim-Uçok et al., 2008b (147) | Acute and Post-acute phase of illness  **P50** difference*  **P50** ratio**  **P50** S1 amplitude  **P50** S2 amplitude  **P50** S1 latency  **P50** S2 latency | FES = 16  HCs = 24 | DSM-IV | *Acute phase*  **P50 difference**  FES > HCs  **P50 ratio**  FES > HCs  *Post-acute phase*  **P50 difference**  **P50 ratio**  **P50 S1 amplitude**  **P50 S2 amplitude**  **P50 S1 latency**  **P50 S2 latency**  No differences  *Comparison between acute and post-acute phases*  **P50 difference**  FES acute > FES post-acute  **P50 ratio**  FES acute > FES post-acute |
| Devrim-Üçok et al., 2016 (216) | **P300** amplitude  **P300** latency | FES = 14  HCs = 22 | BPRS-positive subscale  SCID (DSM-IV) | **P3b amplitude**  FES < HCs  **P3b latency**  No differences |
| Du et al., 2015  (235) | **N400** amplitude  **N400** latency | FES = 58  HCs = 62 | DSM-IV | **N400 latency**  FES > HCs  **N400 amplitude**  FES < HCs |
| Düring et al., 2014 (155) | **P50** S1 amplitude  **P50** S2 amplitude  **P50** ratio** | FES = 61  HCs = 47 | SCAN  ICD-10 | **P50 S1/S2/ratio**  No differences |
| Düring et al., 2016 (200) | **dMMN** amplitude  **pMMN** amplitude  **P3a** amplitude | FES = 51  HCs = 48 | ICD-10 | **dMMN amplitude**  No differences  **pMMN amplitude**  No differences  **P3a amplitude**  FES < HCs |
| Ebdrup et al., 2019 (158) | Machine learning model with EEG- indices (**P50** amplitude and gating; **pMMN**, **dMMN** amplitude) | FES = 46  HCs = 58 | ICD-10 | **ML Model**  Accuracies of P50 and/or MMN in the model to distinguish FES from HCs did not exceed chance level. |
| Frommann et al., 2008 (169) | **P300** amplitude  **P300** latency  **N100** amplitude | EIPS = 50  LIPS = 50  HCs = 40 | BSABS | **P300 amplitude**  LIPS < HCs  EIPS < HCs  EIPS = LIPS  **P300 latency**  No differences  **N100 amplitude**  No differences |
| Fusar-Poli et al., 2011 (225) | **P3b** amplitude | ARMS = 39 (ARMS-T=10 e ARMS-NT=29)  HCs = 13 | CAARMS | **P3b amplitude**  ARMS < HCs  ARMS-T = ARMS-NT |
| Guo et al., 2014 (217) | **P300** amplitude  **P300** latency | FES = 18  HCs = 17 | ICD-10 | **P300 amplitude**  FES < HCs  **P300 latency**  No differences |
| Haigh et al., 2017 **(Meta-analysis) (65)** | Meta-analysis on **MMN** | FES studies pMMN = 9 studies  dMMN = 11 studies | N.A. | **pMMN amplitude**  FES = HCs  **dMMN amplitude**  FES < HCs |
| Hamilton et al., 2018 (214) | **P3a** amplitude  **P3a** latency  **P3b** amplitude  **P3b** latency | PRS = 43 (PRS-C=15; PRS-NC=28)  SCZ = 19  HCs = 43 | SIPS  SCID (DSM-IV) | **P3a amplitude**  PRS < HCs  SCZ < HCs  SCZ = PRS  **P3b amplitude**  PRS < HCs  SCZ < HCs  SCZ = PRS  PRS-C < PRS-NC  **P3a latency**  No differences  **P3b latency**  No differences |
| Hamilton et al., 2020 (68) | **P3a** amplitude  **P3a** latency  **P3b** amplitude  **P3b** latency | PRS = 552 (PRS-C=73; PRS-NC=479)  HCs = 236 | SOPS | **P3a amplitude**  PRS < HCs  **P3a latency**  No differences  **P3b amplitude**  PRS < HCs  PRS-C < PRS-NC  **P3b latency**  No differences |
| Hassan et al., 2020 (215) | **P300** amplitude  **P300** latency | FEP = 108  HCs = 50 | DSM-IV | **P300 amplitude**  FEP < HCs  **P3a latency**  FEP > HCs |
| Hermens et al., 2010 (188) | **dMMN** amplitude **dMMN** latency **P3a** amplitude **P3a** latency | FEP = 17  HCs = 17 | DSM -IV-TR | **dMMN amplitude**  FEP < HCs  **dMMN latency**  No differences  **P3a amplitude**  FEP < HCs  **P3a latency**  No differences |
| Higuchi et al., 2013 (181) | **dMMN** amplitude | ARMS = 17 (ARMS-C=4; ARMS-NC=13)  SCZ = 11  FES = 20  HCs = 20 | SCID (DSM-IV)  CAARMS | **dMMN amplitude**  FES < HCs  SCZ < HCs  ARMS-C < HCs  ARMS-NC = HCs |
| Higuchi et al., 2014 (206) | **dMMN** amplitude  **dMMN** latency  **P3a** amplitude  **P3a** latency | ARMS = 19  (ARMS-C = 4; ARMS -NC=15)  FES= 19  SCZ = 19  HCs = 19 | SCID (DSM-IV) CAARMS | **dMMN amplitude**  ARMS-C < HCs  ARMS -NC = HCs  ARMS-C < ARMS-NC  **dMMN latency**  No differences **P3a amplitude** No differences **P3a latency** No differences |
| Hirayasu et al., 1998 (231) | **P300** amplitude  **P300** latency | FEP = 47  HCs = 73 | DSM-III-R | **P300 amplitude**  FEP < HCs  **P300 latency**  No differences |
| Hong et al., 2009  (144) | **P50** ratio  **P50** S2 amplitude  **P50** latencies | FES = 65  HCs = 62 | DSM-IV | **P50 ratio**  FES > HCs  **P50 S2 amplitude**  FES > HCs  **P50 latencies**  No differences |
| Hsieh et al., 2012 (151) | **dMMN**  **P50** ratio  **P50** difference  **N100** ratio  **N100** difference  **N100** S1 amplitude  **N100** S2 amplitude | UHR = 30  E-BARS = 37  FEP = 32  HCs = 56 | TP-DIS  DSM-IV  ICD-10  CASIS | **dMMN amplitude**  FEP < HCs  UHR < HCs  E-BARS < HCs  **P50 amplitude**  No differences  **N100 amplitude**  No differences |
| Hsieh et al., 2019 (150) | **N100** difference*  **N100** ratio**  **N100** S1 amplitude  **N100** S2 amplitude  **P50** difference*  **P50** ratio** | UHR = 23  FES = 19  HCs = 120 | CAARMS  DSM-IV | **N100 difference**  CHR+FEP > HCs  FEP > CHR  **N100 ratio**  CHR+FEP > HCs  FEP = CHR  **N100 S1 amplitude**  No differences  **N100 S2 amplitude**  No differences  **P50 difference/ratio**  No differences |
| Jahshan et al., 2012 (201) | **dMMN** amplitude **dMMN** latency | SCZ = 33 recent-onset patients = 31 At-risk subjects = 26 HCs = 28 | SIPS  DSM-IV | **dMMN amplitude** SCZ < HCs  Recent-onset patients < HCs  At-risk subjects < HCs  Recent-onset patients = At-risk subjects  At-risk subjects < SCZ  **dMMN latency** No differences |
| Kaur et al., 2011(189) | **dMMN** amplitude **dMMN** latency  **P3a** amplitude  **P3a** latency | FEP = 35  HCs = 17 | SCID (DSM-IV) | **dMMN amplitude**  FEP < HCs  **dMMN latency**  No differences  **P3a amplitude**  FEP < HCs  **P3a latency**  No differences |
| Koshiyama et al., 2017 (192) | **dMMN** amplitude  **pMMN** amplitude | FEP = 14  UHR = 16  HCs = 16 | SIPS | **dMMN amplitude**  FEP < HCs  UHR < HCs  FEP = UHR  **pMMN amplitude**  No differences |
| Kruiper et al., 2019 (213) | **P3a** amplitude  **P3a** latency  **P3b** amplitude  **P3b** latency | FES = 73  HCs = 95 | SCAN | **P3a amplitude**  FEP < HCs  **P3b amplitude**  FEP < HCs  **P3a latency**  FEP > HCs  **P3b latency**  No differences |
| Lee et al., 2010  (165) | **P300**(**visual)** amplitude  **P300** **(visual)** latency  **N100** amplitude  **N100** latency | UHR = 16  FES = 21  HCs = 16 | SIPS  SCID (DSM-IV) | **P300 amplitude**  FEP < HCs  UHR < HCs  UHR = FES  **P300 latency**  No differences  **N100 amplitude**  No differences  **N100 latency**  Not reported |
| Lepock et al., 2018 **(Systematic Review)** (63) | **Systematic Review** on **P50, N100, MMN, P3a** and **P3b** | CHR = 29 studies | N.A. | N.A. |
| Lepock et al., 2019 (128) | **P3a** amplitude  **N400** effects | CHR = 36  HCs = 22 | SIPS | **P3a amplitude**  CHR < HCs  **N400 effects**  CHR < HCs |
| Lho et al., 2019 (218) | **P300** amplitude  **P300** latency | FES = 24  HCs = 24 | SCID (DSM-IV) | **P300 amplitude**  FEP < HCs  **P300 latency**  No differences |
| Lho et al., 2020 (193) | BL and 1-year FU  **dMMN** amplitude **dMMN** latency | FES = 25  HCs = 25 | DSM-IV | **dMMN amplitude**  FES < HCs  FES-FU < FES-BL  **dMMN latency** FES = HCs FES-FU > FES-BL |
| Luo et al., 2019 (148) | **P50** difference*  **P50** ratio** | FEP = 44  UHR = 34  HR = 19  HCs = 39 | DSM-IV  SIPS-COPS | **P50 difference**  FEP > HCs  UHR > HCs  HR > HCs  **P50 ratio**  FEP > HCs  UHR > HCs  HR > HCs |
| Magno et al. 2008  (182) | **dMMN** amplitude  **pMMN** amplitude | SCZ=45  first-degree unaffected biological relatives = 25  FES=12  HCs=27 | DSM-IV | **dMMN amplitude**  SCZ < FES  SCZ < HCs  SCZ < Relatives  FES = HCs  **pMMN amplitude**  SCZ < FES  SCZ < Relatives  SCZ < HCs  Relativies < HCs  FES = HCs |
| McCarley et al., 2002 (224) | **P300** amplitude  **P300** latency | FEP = 33 (FES=15; BP=18)  HCs = 18 | SCID (DSM-III-R) | **P300 amplitude**  FES < BP&HCs (left temporal site)  **P300 latency**  No differences |
| Monaghan et al., 2019 (166) | **N100** amplitude  **P3a** amplitude  **P3b** amplitude | FEP = 41  HCs = 29 | SCID (DSM-IV) | **N100amplitude/**  **P3a amplitude/**  **P3b amplitude**  No differences |
| Mondragón-Maya et al., 2013 (209) | **pMMN** amplitude  **pMMN** latency  **P3a** amplitude  **P3a** latency | CHR = 23  FEP = 20  HCs = 24 | SCID (DSM-IV)  SIPS | **pMMN amplitude**  No differences  **pMMN latency**  No differences  **P3a amplitude**  FEP < HCs  CHR < HCs  **P3a latency**  No differences |
| Morales-Muñoz et al., 2017 (210) | **pMMN** amplitude  **P300** early amplitude  **P300** late amplitude  **N100** amplitude | FEP = 38  HCs = 38 | SCID (DSM-IV) | **pMMN amplitude**  No differences  **P3 early amplitude**  FEP < HCs  **P3 late amplitude**  FEP < HCs  **N100 amplitude**  No differences |
| Morales-Muñoz et al., 2016 (152) | **P50** difference*  **P50** ratio**  **N100** difference*  **N100** ratio** | FEP = 38  HCs = 38 | SCID (DSM-IV) | **P50 difference**  No differences  **P50 ratio**  No differences  **N100 difference** No differences  **N100 ratio**  FEP > HCs |
| Murphy, T. K. et al., 2020 (183) | **dMMN** amplitude  **pMMN** amplitude | FES = 40  SCZ = 50  HCs FES-paired = 40  HCs SCZ-paired = 50 | SCID (DSM-IV) | **dMMN amplitude**  SCZ< HCs  FES = HCs  **pMMN amplitude**  SCZ< HCs  FES = HCs |
| Myles-Worsley et al., 2004 (149) | **P50** ratio** | GHR = 44  CHR = 43  HCs = 39 | CAARMS | **P50 ratio**  GHR > HCs  CHR > HCs  GHR = CHR |
| Nagai et al., 2013 (194) | **dMMN** amplitude  **pMMN** amplitude **dMMN** latency **pMMN** latency  **P3a** amplitude  **P3a** latency | UHR = 21  FES = 20  HCs = 22 | SIPS  DSM-IV | **dMMN amplitude**  FEP < HCs  UHR < HCs  **pMMN amplitude**  No differences **dMMN latency**  No differences **pMMN latency**  No differences  **P3a amplitude**  FEP < HCs  UHR < HCs  UHR = FEP  **P3a latency**  No differences |
| Nagai et al., 2013 **(Review)** (186) | Review on **MMN** | FES = 9 Studies FEP = 4 Studies | N.A. | N.A. |
| Nagai et al., 2017 (66) | **dMMN** amplitude  **pMMN** amplitude | UHR = 21  FEP = 19  HCs = 16 | SIPS  DSM-IV | **dMMN amplitude**  FEP < HCs  UHR < HCs  FEP = UHR  **pMMN amplitude**  UHR < HCs  FEP = HCs  FEP = UHR |
| Oranje et al., 2013 (145) | **P50** ratio** | FES = 30  HCs = 32 | DSM-IV | **P50 ratio**  FES > HCs |
| Oribe et al., 2013 (161) | **P300 (visual)**  amplitude  **P300 (visual)**  latency  **N100** amplitude  **N100** latency | PRO = 23  FES = 17  HCs = 31 | SIPS  SCID (DSM-IV-TR) | **P300 amplitude**  FEP < HCs  PRO < HCs  PRO = FEP  **P300 latency**  FEP > HCs  PRO > HCs  PRO = FEP  **N100 amplitude**  FEP < HCs  **N100 latency**  No differences |
| Oribe et al., 2020 (170) | **P300 (visual)**  amplitude  **P300 (visual)**  latency  **N100** amplitude  **N100** latency | CHR = 19  HCs = 28 | SIPS | **P300 amplitude**  CHR < HCs  **P300 latency**  CHR > HCs  **N100 amplitude**  No differences  **N100 latency**  No differences |
| Özgürdal et al., 2008 (226) | **P300** amplitude | PP = 54  FEP = 31  SCZ = 27  HCs = 54 | SOPS  PANSS  ICD-10  SCID (DSM-IV) | **P300 amplitude**  PP < HCs  FEP < HCs  SCZ < HCs |
| Perez et al., 2014 (203) | **dMMN** amplitude  **pMMN** amplitude | SCZ = 19  CHR = 38 (CHR-NT = 15; CHR-T = 16; 7 drop out)  HCs = 44 | COPS  SIPS | **dMMN, pMMN amplitude**  CHR < HCs  SCZ < HCs  CHR-C < CHR-NC |
| Qiu et al., 2014 **(Meta-analysis)** (67) | **Meta-analysis** on **P300** | 17 Studies included FES subjects | N.A. | **P300 amplitude**  FES< HCs  **P300 Latency**  FES > HCs |
| Randau et al., 2019 (195) | **dMMN** amplitude  **pMMN** amplitude  combined **MMN**  **P3a** amplitude  **P3a** latency | FEANS = 56  HCs = 64 | ICD-10 | **dMMN** amplitude  FEANS < HCs  **pMMN** amplitude No differences  **combined MMN**  No differences  **P3a amplitude**  No differences  **P3a latency**  No differences |
| Renoult et al., 2007 (219) | **P300** amplitude  **P300** latency | FES = 27  HCs = 14 | SCID (DSM-IV) | **P3b amplitude**  FEP < HCs  **P3b latency**  No differences |
| Rydkjær et al., 2017 (190) | **dMMN** amplitude  **pMMN** amplitude  combined **MMN**  **P3a** amplitude | FEP = 27 (FES = 13)  HCs = 43 | DSM-IV-TR ICD-10  K-SADS-PL | **dMMN amplitude, pMMN amplitude**  FEP < HCs  FES < HCs  FES < HCs **Combined MMN** No differences **P3a amplitude** No differences |
| Salisbury et al., 1998 (220) | **P300** amplitude | FES = 14  FEAD = 14  HCs = 14 | SCID (DSM-III-R) | FES < HCs  FES < FEAD (*left temporal site*) |
| Salisbury et al., 2017 (211) | **dMMN** amplitude  **pMMN** amplitude | FEP = 29  HCs = 40 | SCID | **pMMN**  No differences  **dMMN**  No differences |
| Salisbury et al., 2018 (187) | Complex **MMN** | Experiment 1  SCZ = 20  HCs = 22  Experiment 2  SCZ = 24  HCs = 21  FES = 21 | SCID (DSM-IV) | **Complex MMN** Experiment 1  SCZ< HCs  Experiment 2  FES = HCs |
| Salisbury et al., 2019 (164) | **pMMN** amplitude  **P3a** amplitude  **N100** amplitude | FES = 106  HCs = 114 | SCID (DSM-IV) | **pMMN amplitude**  No differences  **P3amplitude**  FEP < HCs  **N100 amplitude**  FEP < HCs |
| Salisbury et al., 2020a (197) | **dMMN** amplitude  **pMMN** amplitude  Complex **MMN** | FES = 22  HCs = 22 | SCID (DSM-IV) | **dMMN amplitude, pMMN amplitude**  No differences  **Complex MMN**  FES < HCs |
| Salisbury et al., 2020b (198) | **dMMN** amplitude  **pMMN** amplitude  **dMMN** latency  **pMMN** latency | FES = 27  HCs = 27 | SCID (DSM-IV) | **pMMN amplitude**  FES < HCs  **dMMN amplitude**  No differences  **dMMN latency**  No differences  **pMMN latency**  No differences |
| Shaikh et al., 2012 (202) | **dMMN** amplitude | ARMS = 41 (ARMS-C=10; ARMS-NC=31)  HCs = 50 | CAARMS  SCID (DSM-IV) | **dMMN amplitude**  ARMS < HCs  ARMS-C < ARMS-NC |
| Solís-Vivanco et al., 2014 (191) | **dMMN** amplitude | FEP = 20  UHR = 20  HCs = 23 | SCID (DSM-IV)  SIPS | **dMMN amplitude**  FEP < HCs  UHR < HCs |
| Song et al., 2014 (146) | **P50** S2 amplitude  **P50** S2 latency  **P50** ratio** | FES = 49  HCs = 43 | CCMD-3  DSM-IV | **P50 S2 amplitude**  FES > HCs  **P50 S2 latency**  No differences  **P50 ratio**  FES > HCs |
| Tang et al., 2019 (228) | **P300** oddball amplitude  **P300** oddball latency  **P300** novel amplitude  **P300** novel latency | P300 Odball paradigm  CHR = 104 (CHR-C=19; CHR-NC=85; CHR-rem=53; CHR  notRem=32)  HCs = 69  P300 Novel paradigm  CHR = 131 (CHR-C=23; CHR-NC=108; CHR-rem=68; CHR-notRem=40)  HCs = 69 | SIPS | **P3 oddball amplitude**  No differences  **P3 novel amplitude**  CHR-C < HCs  CHR-C < CHR-NC  CHR-NC = HCs  CHR-C < CHR-rem  CHR- notRem < HCs  CHR- notRem < CHR-rem  **P300 oddball latency**  No differences  **P300 novel latency**  No differences |
| Umbricht et al., 2006 (199) | **dMMN** amplitude  **pMMN** amplitude  **P300** amplitude | FES =26  Recent onset SCZ = 25  SCZ = 25  HCs = 39 | RDC  SCID (DSM-II-R) | **pMMN amplitude**  Recent onset < HCs  SCZ < HCs  FES = HCs  **dMMN amplitude**  SCZ < HCs  FES = HCs  **P300 amplitude**  FES < HCs  SCZ < HCs |
| Valkonen-Korhonen et al., 2012 (167) | **N100** amplitude  **N100** latency  **P300** amplitude  **P300** latency | FEP = 7  HCs = 7 | Not specified | **N100 amplitude/latency**  No differences  **P300 amplitude/ latency**  No differences |
| van der Stelt et al., 2005 (227) | **P300** amplitude  **P300** latency | Putatively Prodromal = 10  FES = 10  SCZ = 14  HCs younger = 14  HCs older = 14 | DSM-IV | **P300 amplitude**  Putatively Prodromal < HCs  FEP < HCs  SCZ < HCs  Putatively Prodromal = FEP = SCZ  **P300 latency**  No differences |
| van Tricht et al., 2010 (171) | **P300** amplitude  **N100** amplitude | UHR = 61 (UHR-T=18; UHR-NT=43)  HCs = 28 | SIPS | **P300 amplitude**  UHR-T < HCs  UHR-T < UHR-NT  UHR-NT < HCs  **N100 amplitude**  No differences |
| van Tricht et al., 2015 (156) | **P50** ratio** | UHR = 61  HCs = 28 | SIPS  PANSS | **P50 ratio**  No differences |
| Wang et al., 2003 (223) | **P300** amplitude  **P300** latency | FES = 20  HCs = 23 | DSM-IV | **P300 amplitude**  FEP < HCs  **P300 latency** No differences |
| Wang et al., 2010 (222) | **P300** amplitude  **P300** latency | FES = 19  HCs = 25 | ICD-10 | **P300 amplitude**  FEP < HCs  **P300 latency**  No differences |
| Xiong et al., 2010(221) | **P300** amplitude  **P300** latency | FES = 30  HCs = 28 | DSM-IV | **P300 amplitude**  FEP < HCs  **P300 latency**  No differences |
| Xiong et al., 2019(95) | **dMMN** amplitude  **pMMN** amplitude | FES = 40  SCZ = 40  HCs = 40 | SCID (DSM-IV) | **dMMN amplitude**  FES < HCs  SCZ < HCs  **pMMN amplitude**  SCZ < HCs |
| Xu et al., 2016 (172) | **P300** amplitude  **P300** latency  **N100** amplitude  **N100** latency | FES = 16  HCs = 16 | ICD-10 | **P300 amplitude**  FEP < HCs  **P300 latency**  No differences  **N100 amplitude**  FEP < HCs  **N100 latency**  No differences |
| Yee et al., 2010 (153) | **P50** ratio**  **P50** amplitude | Recent-onset = 16 (within three years of their first psychotic episode)  SCZ = 12  HCs=28 | DSM-IV | **P50 ratio**  SCZ > HCs  Recent-onset = HCs  Recent-onset = SCZ  **P50 amplitude**  No differences |
| Ziermans et al., 2012 (157) | **P50** ratio** | UHR = 63  HCs = 68 | COGDIS  SIPS  BSABS | **P50 ratio**  No differences |

* Difference is defined as S2-S1 amplitude; **Ratio is defined as S2/S1 amplitude

All the studies that analysed P300/P3a/P3b used auditory paradigms except references (161),(165) and (170).

At Risk (AR); AR who converted (AR-C); AR who did not convert (AR-NC); At Risk Mental State (ARMS); ARMS who converted to psychosis (ARMS-C); ARMS who did not convert to psychosis (ARMS-NC); ARMS who did not transition (ARMS-NT); ARMS who did transition (ARMS-T); duration deviant Mismatch Negativity (dMMN); Brief Psychiatric Rating Scale (BPRS); Bonn Scale for the Assessment of Basic Symptoms (BSABS); Comprehensive Assessment of At Risk Mental States (CAARMS); Chinese Classification of Mental Disorders (CCMD); Clinical High Risk (CHR); CHR who converted to psychosis (CHR-C); CHR who did not convert to psychosis (CHR-NC); CHR not in remission (CHR-notRem); CHR who did not transition (CHR-NT); CHR in remission (CHR-rem); CHR who did transition (CHR-T); high-risk criterion Cognitive Disturbances (COGDIS); at-risk criterion Cognitive-Perceptive Basic Symptoms (COPER); Criteria of Prodromal Syndromes (COPS); Diagnostic and Statistical Manual of Mental Disorders (DSM); Early/Broad At-Risk mental States (E-BARS); Early Initial Prodromal States (EIPS); Early Recognition Inventory based on the retrospective assessment of the onset of schizophrenia (ERIraos); First Episode Antipsychotic-Naïve Schizophrenia (FEANS); First-Episode Psychosis (FEP); First-Episode Schizophrenia (FES); First-Episode Schizophrenia Acute phase (FES-A); First-Episode Schizophrenia Post-Acute phase (FES-PA); Genetically High-Risk (GHR); Healthy Controls (HCs); International Statistical Classification of Diseases (ICD); Kiddie-SADS-Present and Lifetime Version (K-SADS-PL); Late Initial Prodromal States (LIPS); Low Resolution Electromagnetic Tomography (LORETA); Positive and Negative Syndrome Scale (PANSS); pitch deviant Mismatch Negativity (pMMN); Prodromal Patients (PP); Prodromal (PRO); Psychosis Risk Syndrome (PRS); Psychosis Risk Syndrome who converted to psychosis (PRS-C); Psychosis Risk Syndrome who did not convert to psychosis (PRS-NC); Schedules for Clinical Assessment in Neuropsychiatry (SCAN); Structured Clinical Interview for the Diagnostic and Statistical Manual of Mental Disorders (SCID); Structured Clinical Interview for the Positive and Negative Symptom Scale (SCI-PANSS); Chronic Schizophrenia (SCZ); Structured Interview of Psychosis-risk Syndromes (SIPS); Structured Interview for Psychosis-Risk Syndrome, Criteria of Psychosis-risk Syndromes (SIPS-COPS); Scale of Psychosis-Risk Symptoms (SOPS); Schizophrenia Proneness Instrument, Adult Version (SPI-A); Thought/Perception Diagnostic Interview Schedule (TP-DIS); Ultra High Risk (UHR); UHR who did not transition (UHR-NT); UHR who did transition (UHR-T).
